# Dual‐Locking the SARS‐CoV‐2 Spike Trimer: An Amphipathic Molecular “Bolt” Stabilizes Conserved Druggable Interfaces for Coronavirus Inhibition

**DOI:** 10.1002/advs.202417534

**Published:** 2025-04-26

**Authors:** Shiliang Li, Fang Ye, Yucheng Zheng, Jie Wang, Haoran Peng, Lili Zhu, Lili Chen, Tao Yu, Huan Ge, Jiaqi He, Binghao Zhang, Jiayun Wu, Zhiyi Zhang, Liangliang Jiang, Geng Chen, Ping Zhao, Ke Lan, Zhenjiang Zhao, Xuhong Qian, Ke Xu, Yang Du, Honglin Li

**Affiliations:** ^1^ Innovation Center for AI and Drug Discovery School of Pharmacy East China Normal University Shanghai 200062 China; ^2^ Shanghai Key Laboratory of New Drug Design School of Pharmacy East China University of Science & Technology Shanghai 200237 China; ^3^ Department of Pain Management HuaDong Hospital affiliated to Fudan University Shanghai 200040 China; ^4^ Kobilka Institute of Innovative Drug Discovery School of Medicine Chinese University of Hong Kong Shenzhen Guangdong 518172 China; ^5^ State Key Laboratory of Virology and Biosafety Taikang Center for Life and Medical Sciences College of Life Sciences Wuhan University Wuhan Hubei 430072 China; ^6^ Department of Microbiology Navy Medical University Shanghai 200433 China; ^7^ Institute of Interdisciplinary Integrative Medicine Research Shanghai University of Traditional Chinese Medicine Shanghai 201203 China; ^8^ Lingang Laboratory Shanghai 200031 China

**Keywords:** amphipathic molecular bolt, druggable interfaces, entry inhibitor, RBD‐NTD interface, RBD‐RBD interface, SARS‐CoV‐2, spike trimer stabilization

## Abstract

The SARS‐CoV‐2 spike (S) protein, a trimeric structure comprising three receptor binding domains (RBDs) and three N‐terminal domains (NTDs), undergoes substantial conformational changes to a fusion‐prone open state for angiotensin‐converting enzyme 2 (ACE2) binding and host cell infection. Stabilizing its closed state is a key antiviral strategy but remains challenging. Here, we introduce S416, a novel amphipathic molecule acting as a “molecular bolt”. Cryo‐EM study reveals that S416 binds concurrently to six sites across two distinct druggable interfaces: three molecules at the RBD‐RBD interfaces and three at the NTD‐RBD interfaces. This unique “dual‐locking” mechanism, driven by S416's polar carboxyl head and nonpolar phenylthiazole tail, robustly stabilizes the spike trimer in a locked, closed conformation through strong inter‐domain interactions, reducing structural flexibility and atomic fluctuations compared to the apo structure resolved synchronously. Crucially, these RBD‐RBD and NTD‐RBD interfaces are conserved across human‐infecting coronaviruses, suggesting potential as broad‐spectrum antiviral targets. Our findings demonstrate that the highly dynamic spike trimer can be effectively stabilized by an amphipathic molecular bolt targeting both the inter‐ and intra‐monomer interfaces, offering a promising strategy against emerging coronaviruses.

## Graphical abstract

A new amphipathic molecule, S416 is discovered, that locks the SARS‐CoV‐2 spike protein in its closed state, blocking viral entry. S416 acts as a molecular bolt, binding six sites: three between adjacent RBDs and three connecting NTDs to RBDs. This dual‐locking mechanism stiffens the spike structure and reduces its flexibility. Targeted interfaces are shared across coronaviruses, suggesting S416‐inspired drugs could broadly inhibit current and emerging variants.

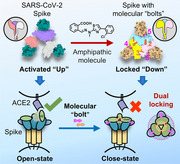

## 1 Introduction

The global health crisis triggered by the SARS‐CoV‐2 pandemic has underscored the urgent need for effective therapeutic strategies against COVID‐19. At the forefront of this quest is the S protein, a trimeric glycoprotein that mediates the critical first step of viral entry into host cells.^[^ ^1^ ^]^ The S protein's structure and functionality are central to its role in binding to the human ACE2 receptor, making it an ideal target for intervention strategies.^[^ ^2^ ^]^

Despite the significant advances in understanding the S protein, limitations remain in our ability to target it effectively.^[^ ^3^ ^]^ The receptor‐binding domain (RBD) of the S protein has been extensively studied due to its direct involvement in ACE2 interaction.^[^ ^4^ ^]^ This region exists in a dynamic equilibrium, going from a “closed” (“down”) conformation to a receptor accessible “open” (“up”) conformation to bind ACE2, thus triggering cellular entry.^[^ ^5^ ^]^ However, the emergence of SARS‐CoV‐2 variants with mutations in the RBD has highlighted the vulnerability of RBD‐targeted drugs and vaccines to resistance, necessitating the exploration of additional regions to target in the S protein.^[^ ^6^ ^]^ The N‐terminal domain (NTD) of the S protein, while not directly involved in ACE2 binding, is recognized for its contribution to the overall conformation and stability of the S protein.^[^ ^7^ ^]^ The potential of the NTD as a therapeutic target site has been relatively underexplored, but its role in the S protein's structure suggests that it could provide an opportunity for innovative approaches to stabilizing the S protein and impeding viral entry.^[^ ^8^ ^]^

In this study, we reported the first locked structure of the SARS‐CoV‐2 spike trimer tightly stabilized by six identical small molecules binding at the RBD‐RBD and RBD‐NTD interfaces. These binding interfaces, which are largely conserved among human‐infecting coronaviruses, offer a promising avenue for the development of broad‐spectrum drugs against emerging coronavirus strains. The innovative “dual‐locking” approach to the S protein trimer presents a novel strategy, potentially fortifying our defenses against the virus and its evolving variants. This strategy signifies a promising direction for future research and therapeutic development.

## 2 Results

### 2.1 Discovery of a Novel Locker for the SARS‐CoV‐2 Spike Protein through Targeting the RBD‐RBD and RDB‐NTD Interfaces

**Figure 1:**
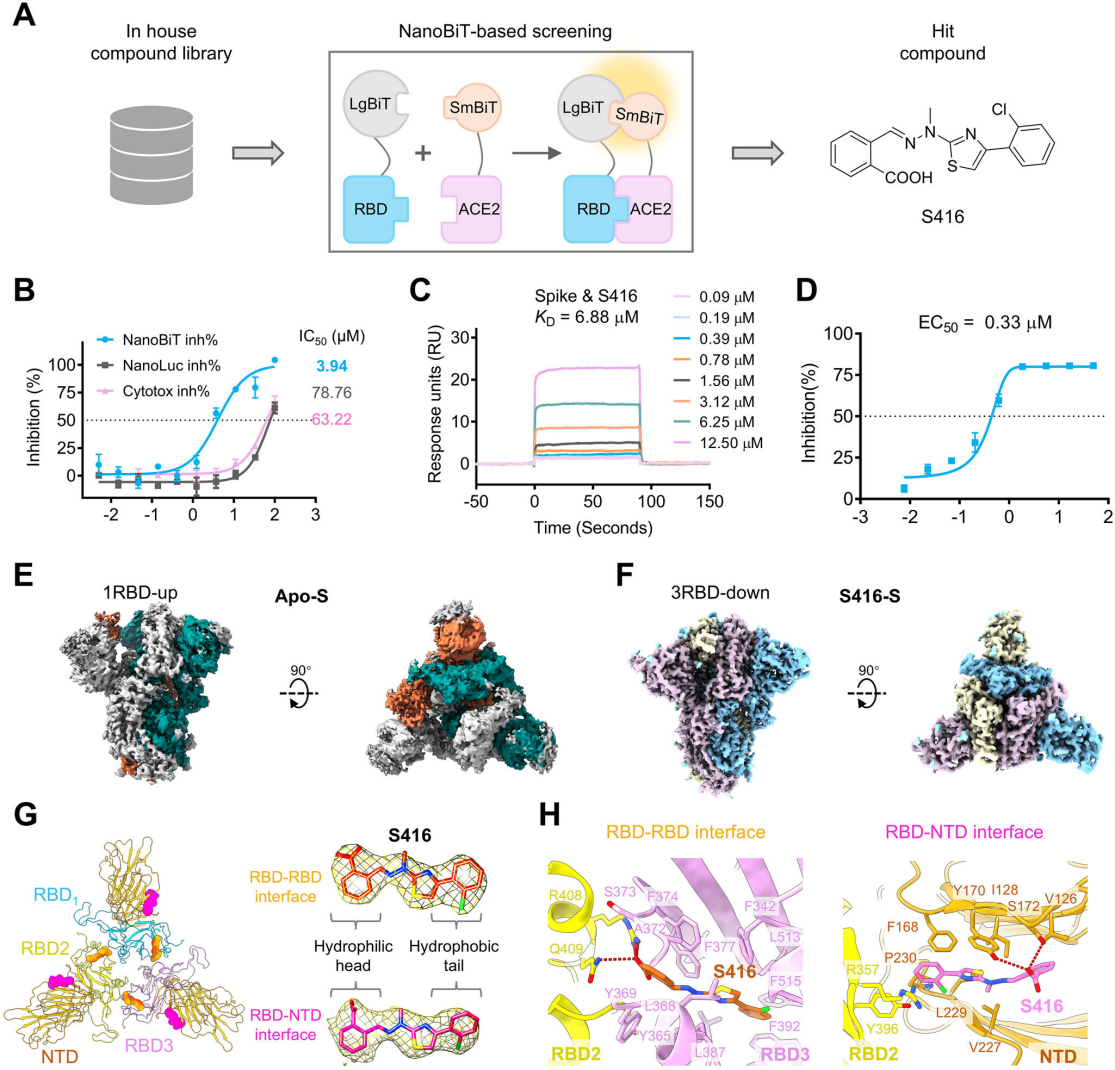
Discovery of a novel locker for the SARS‐CoV‐2 spike protein through targeting the RBD‐RBD and RBD‐NTD interfaces. A) NanoBiT‐based screening of the in house compound library to identify a potent DHODH inhibitor S416 interrupting the interaction of SARS‐CoV‐2 S‐RBD/ACE2. B) The potency of S416 inhibiting the recognition of RBD/ACE2 in the NanoBiT assay. NanoBiT inh%: the inhibition rates against SARS‐CoV‐2 S‐RBD/ACE2 interaction; NanoLuc inh%: the inhibition rates against NanoLuc luciferase; Cytotox inh%: the inhibition rates against the transfected HEK293 cell proliferation. A compound's Cytotox inh% has a positive impact on its NanoLuc inh%; however, this relationship does not hold in reverse. C) SPR analysis of the binding affinity for S416 with the trimeric spike protein. D) SARS‐CoV‐2‐S pseudotyped particle entry assay to evaluate S416's potency in inhibiting cellular entry. Data are presented as mean ± SD from triplicate measurements. E) Cryo‐EM map of apo SARS‐CoV‐2 spike trimer in an open state. F) Cryo‐EM map of S416‐bound SARS‐CoV‐2 spike trimer in a tightly locked state. G) Three S416 molecules (orange) bind at the RBD‐RBD interfaces (RBD‐pockets) and another three S416 molecules (pink) bind at the RBD‐NTD interfaces (NTD‐pockets). S416 fit cryo‐EM densities are shown as mesh. H) S416 and its interacting residues in the RBD‐RBD and RBD‐NTD pockets.

To identify antiviral therapeutics blocking SARS‐CoV‐2 cellular entry, a NanoBiT‐based screening for inhibitors disrupting the SARS‐CoV‐2 S‐RBD/ACE2 interaction was conducted against our in‐house compound library (**Figure** **1**A). The most exciting finding was that a compound with hydrazino‐thiazole scaffold, named S416, could inhibit the interaction of SARS‐CoV‐2 S‐RBD and ACE2 (NanoBiT inh%) at low concentrations (IC_50_ = 3.94 µm, Figure 1B). An ≈20‐fold less potent activity to inhibit the NanoLuc luciferase (NanoLuc inh%) supported that S416 is not a false positive hit. In a drug affinity responsive target stability (DARTS) assay, we observed that S416 could protect RBD from being digested by pronase (Figure S1A, Supporting Information). This phenomenon gave us a clue that S416 may interact with RBD to block the recognition of RBD/ACE2. 1H CPMG NMR and saturation transfer difference (STD) NMR experiments were then conducted and confirmed that S416 could indeed bind to RBD directly (Figure S1B,C, Supporting Information). The binding affinity of S416 to the trimeric spike protein was immediately determined by surface plasmon resonance (SPR) assay, and the result showed that S416 could bind to the spike protein with a *K*_D_ value of 6.88 µm (Figure 1C). In the SARS‐CoV‐2‐S pseudotyped particle entry assays, S416 significantly inhibited the SARS‐CoV‐2‐S pseudovirus attachment to ACE2‐expressing HEK293T cells with the EC_50_ of 0.33 µm (Figure 1D). Further, we tested the effect of S416 on cell viability in the pseudotyped particle entry assay, and obtained a CC_50_ of 436.30 µm, leading to a high selectivity index (SI) of 1322. The particle size analysis of S416, conducted using dynamic light scattering (DLS), coupled with its inhibitory effects on the counter‐screening enzymes MDH and AmpC, confirmed that S416 does not possess colloidal properties in vitro^[^ ^9^ ^]^ (Figure S1D–F, Supporting Information).

To further investigate how S416 binds to the SARS‐CoV‐2 spike trimer, we used the cryo‐EM technique. Based on the reported genome sequence of SARS‐CoV‐2, we expressed the ectodomain residues 14 to 1208 of spike protein with a C‐terminal 6×His tag and purified the spike ectodomain from sf9 cells by affinity chromatography and size‐exclusion chromatography (Figure S2A, Supporting Information). Then, the spike protein with or without S416 was prepared for the cryo‐EM grids. Cryo‐EM data for both the apo‐spike and S416‐spike were collected and processed with cryo‐sparc v4.0.2 (Figures S2 and S3, Supporting Information). Focused on 3D classification, ≈87% (deduct other particles) apo‐spike particles were in the open conformation, with a single RBD “up” and two RBDs “down” position based on the 2.86 Å map (Figure 1E; Figure S2D, Supporting Information, Apo‐S). Interestingly, for S416‐spike, ≈68% of particles were performed on three RBDs “down” positions, while ≈32% were other particles, and no particles were observed in RBD “up” state (Figure 1F; Figure S3C, Supporting Information). After three rounds of refinements, the 2.95 Å map was calculated for the S416‐spike, clearly defining the density of the closed RBDs (Figure 1F, S416‐S).

We were astonished to discover six additional stout densities near the interaction interfaces among the adjacent RBD‐RBD and NTD‐RBD domains. These densities could only be observed in the S416‐spike map, and S416 could be well fitted to the stout density (Figure 1G). Specifically, three S416 molecules bind to the pocket formed by the RBD and the adjacent RBD (named the RBD‐pocket), and the other three bind to the pocket formed by the NTD domain and the adjacent RBD (named the NTD‐pocket). The six S416 binding pockets in the spike protein shared similar characteristics: one hydrophobic region and one polar region, constituting a relatively positive electrostatic surface (Figure S4, Supporting Information).

For the RBD‐pocket, S416's long phenylthiazole head is embedded in a hydrophobic cavity in one RBD, with the carboxyl tail exposing outside the cavity to contact the neighboring RBD (Figure 1H). The phenylthiazole head in the hydrophobic cavity has π‐π stacking interactions with Y369 and F392, forming hydrophobic interactions with surrounding residues Y365, L368, A372, F374, F377, and L387. At the same time, the carboxyl tail interacts with R408 and Q409 from a neighboring RBD through charge‐assisted hydrogen‐bonded networks (Figure 1H, left).

Similarly, for the NTD‐pocket, S416's carboxyl tail interacts with Y170 and S172 through hydrogen‐bonded interactions in NTD, with its phenylthiazole head in the hydrophobic cavity forming hydrophobic interactions with surrounding residues V126, I128, V227, F168, L229 in NTD and Y396 in neighboring RBD (Figure 1H, right). Besides, the chlorophenyl moiety can form cation‐π interaction with R357 to enhance the interactions between RBD and NTD domains. The above data provide solid evidence that S416 is a novel inhibitor of the SARS‐CoV‐2 spike protein by targeting the RBD‐RBD and RBD‐NTD interfaces.

### 2.2 Six Molecular “bolts” Stabilize Closed State of the Spike by Locking the Adjacent Monomers

Analysis of the conformational space of the published spike structures revealed that the features of S416‐spike are similar to the previously reported structures (Figure S5, Supporting Information), in which all RBD domains are in “down” position and compacted tightly. In contrast, in the apo‐spike, one of the RBD domains is in “up” position, which resembles the earliest reported structures like 6VSB. Therefore, the significant difference between S416‐spike and apo‐spike is that the RBDs changed from one “up” to totally “down”.

**Figure 2:**
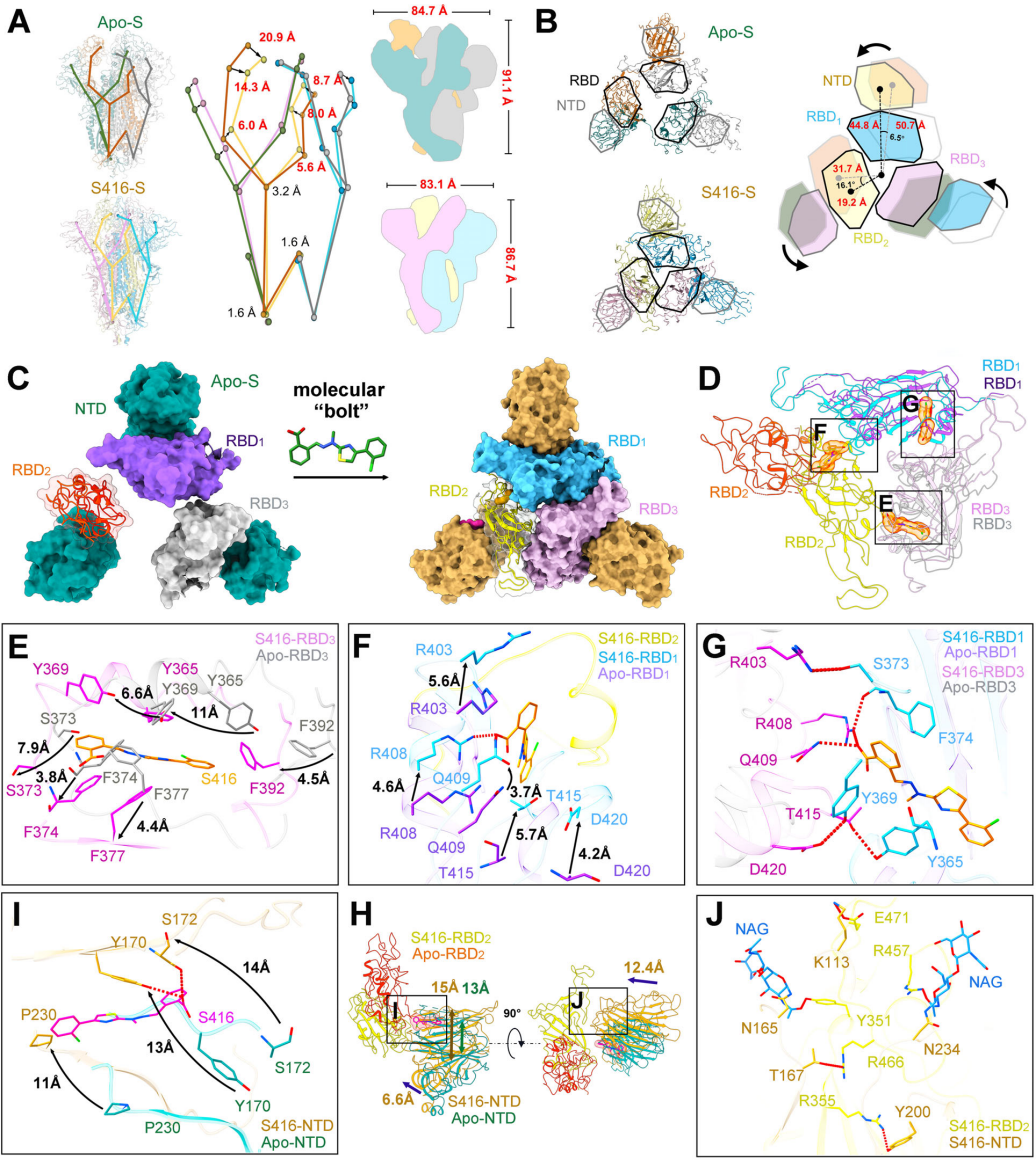
Six molecular “bolts” stabilize closed state of the spike by locking the adjacent monomers. Superimposition of S416‐bound SARS‐CoV‐2 spike trimer (S416‐S) with apo SARS‐CoV‐2 spike trimer (Apo‐S) in a front view with twenty‐seven domain core beads representations of cryo‐EM structures resolved in our study A) or in a top view with RBDs boxed in black and NTD boxed in gray B), and comparison of the difference of RBD and NTD conformations in S416‐S and Apo‐S to highlight the compaction of RBDs and NTDs in the S416‐bound spike structure. Vectors connecting each region's Cα centroids were generated. Core angles were used to define the relative positions of the domains, and the core distances were used to identify the width of differences in domain positioning. Core angles and distances were compared between S416‐spike and apo‐spike. C) One RBD up apo SARS‐CoV‐2 spike trimer (Apo‐S: NTD, dark cyan; RBD1, medium purple; RBD2, coral; RBD3, dark gray) is modulated by S416 to the three RBD download closed conformation (S416‐S: NTD, dark goldenrod; RBD1, deep sky blue; RBD2, yellow; RBD3, thistle). The structures were shown in surface representation. D–G) Alignment of Apo‐S and S416‐S structures to display the details of structural changes focusing on RBDs. E–G) S416 (orange) and key residues are shown as sticks. S416‐RBD and Apo‐RBD are shown as cartoon. Arrows indicated remarkable conformational changes. The hydrogen bonds are shown as red dashed lines. I,J) Alignment of Apo‐S and S416‐S structures to display the details of structural changes focusing on NTDs. S416 (pink) and key residues were shown as sticks. S416‐RBD2 (yellow), S416‐NTD (dark goldenrod) and Apo‐NTD (dark cyan) were shown as cartoon. Arrows indicated remarkable conformational changes. The hydrogen bonds were shown as red dashed lines.

We proposed a quantitative definition of relative positions in specific domains to explore the primary influence of S416 binding on the unique arrangement of distinct domains. A detailed examination of the S416‐spike structure and the apo‐spike structure revealed the following findings (**Figure** **2**A,B). As for the front view (Figure 2A), domains in the S1 subunit (NTD, RBD and subdomains) move as rigid bodies and display a pronounced array of relative shifts between the S416‐spike structure and the apo‐spike structure synchronously resolved in this study. In detail, RBD moved inward by 14–21 Å, SD1 moved inward by 6 Å, and NTD moved inward by 5–9 Å in S416‐spike compared with the apo‐spike, reducing the overall height and width of spike trimer from 91.1 and 86.7 Å to 84.7 and 83.1 Å. As for the top view (Figure 2B), the spike protein underwent an anticlockwise twist from the one RBD “up” conformation to the three RBDs “down” conformation when S416 bound to the Spike protein, which tightened the RBD and NTD domains by decreasing the distance between the individual domain and the central axis. Compared with the apo‐spike, the distance from RBDs core to the central axis is shortened by ≈12 Å, while the distance from NTD core to the central axis is shortened by ≈6 Å. The above results demonstrated that S416 stabilizes the three RBD “down” conformation of the SARS‐CoV‐2 spike trimer by tightening the domains to significantly enhance the interactions between different monomers.

The concurrently binding of six S416 induced the apo SARS‐CoV‐2 spike trimer to transform from one RBD “up” open state to all RBDs “down” close state without any spike particles on the open conformation (Figure 2C; Figure S3, Supporting Information) mainly by enhancing the RBD‐RBD (Figure 2D–G) and NTD‐RBD inter‐domain interactions (Figure 2I,J; Figure S6, Supporting Information). Compared to the Apo‐S structure determined synchronously in this study, rotation and movement of RBDs in S416‐S were detected, tightening the whole RBD domain. Through hydrophobic interaction between S416 and non‐polar residues (F392, etc.), residues on RBD3 deflected to RBD2, resulting in the movement of F377 (4.4 Å), F392 (4.5 Å), Y365 (11 Å), Y369 (6.6 Å), S373 (7.9 Å), and F374 (3.8 Å). Deflection of residues enhanced interactions between RBD3 and other RBDs, where Y365 and Y369 played an important role^[^ ^10^ ^]^ (Figure 2E). S416's strong polar interactions with residues R408 and Q409 in the neighboring RBD1 induces the shift of R403 (5.6 Å), Q409 (3.7 Å), R408 (4.6 Å), T415 (5.7 Å), and D420 (4.2 Å) (Figure 2F). The distance between RBD1 and RBD3 is shortened mainly due to hydrogen bonds generated between RBDs and S416 (R403‐S373, R408‐S416‐F374, T415‐Y365, D420‐Y369) (Figure 2G). Thus, the hydrogen bond networks appeared to play a central role in tightening RBDs, further stabilizing the locked state of the Spike protein. Although the RBD domains become compact after S416 binding, the volume of the inner RBD‐pocket is increases more than twice (320 Å^3^ vs 771 Å^3^) (Figure S6A, Supporting Information). The presence of S416 also stabilized the flexible RBM (core ACE2 binding region), suggesting the ability to inhibit ACE2 binding (Figure S6B, Supporting Information).

Though RBD‐bound S416 tightened RBD conformation through the movement of 4.0 Å (Figure S6E, Supporting Information), NTD‐bound S416 promoted NTD approaching RBD ≈12.4 Å, collectively making NTD and RBD closer (Figure 2H; Figure S6E, Supporting Information). The height of S416 bound NTD‐pocket increased from 13 to 15 Å by the upward movement of NTD upper region residues (Figure 2H). However, the width of the NTD‐pocket is reduced after S416 binding, resulting in a decreased pocket volume of ≈100 Å^3^ (Figure S6C, Supporting Information). S416 binding also stabilized the structure of M1 (14‐ 26), M2 (67‐80), M3 (144‐164), M4 (173‐185), and M5 (243‐253) at the NTD region, whose electron densities are lacked in the Apo‐S (Figure S6D, Supporting Information). Compared to Apo‐NTD, residues on S416‐NTD deflected, including the movement of F230 (11 Å), Y170 (13 Å), and S172 (14 Å) (Figure 2I). S416 binding promoted hydrogen bond networks between NTD and RBD2, including K113‐E471, Y351‐NAG‐N165, R466‐T167, R355‐Y200, and R457‐NAG‐N234. This hydrogen network enabled NTD to get closer to RBD, stabilizing the close state of RBD2 (Figure 2J). The same conformational and interactional changes trend was also observed in the NTD‐RBD3 interface (Figure S6E–H, Supporting Information). The structural changes enhanced the interactions between different monomers, especially the interactions between RBD‐RBD and RBD‐NTD domains, thus stabilizing the locked state of SARS‐CoV‐2 spike trimer (Figure S7, Supporting Information). We, therefore, propose that each of the six S416 worked like a molecular “bolt” to fix the flexible spike trimer to a stable, compact, and rigid locked state by enhancing the RBD‐RBD and NTD‐RBD inter‐domain interactions.

### 2.3 The Reduced Conformational Dynamics Resulted from the Dual‐Locking Mechanisms at the Intermonomeric RBD‐RBD and RBD‐NTD Interfaces

**Figure 3:**
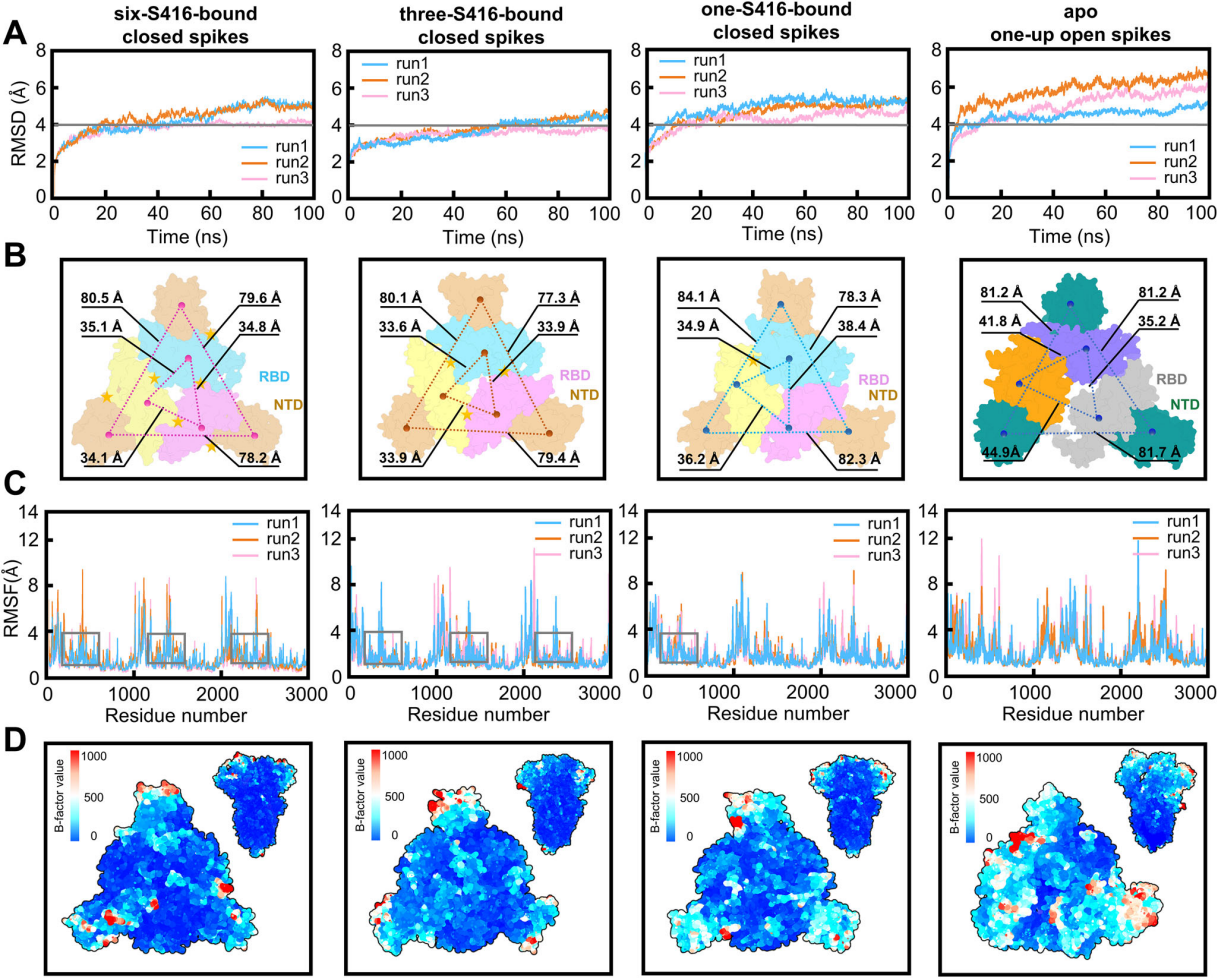
The reduced molecular dynamics and atomic fluctuations when spike trimer is double locked at the intermonomeric RBD‐RBD and RBD‐NTD interfaces. A) The RMSD of six‐, three‐, and one‐ S416‐bound closed, and apo one‐up open SARS‐CoV‐2 spike trimer during the 100 ns for three trajectories. The horizontal gray bars at 4 Å are reference lines designed to visually emphasize the differences in RMSD values across the systems. B) Analysis of the adjacent RBD and NTD core distance of the average conformation in the SARS‐CoV‐2 spike trimer simulating systems (left to right: six‐, three‐, and one‐ S416‐bound closed, and apo one‐up open system), beads indicated centra of mass of RBDs and NTDs, yellow pentagram indicates S416 binding site. C) The RMSF of the SARS‐CoV‐2 spike trimer simulating systems (left to right: six‐, three‐, and one‐ S416‐bound closed, and apo one‐up open system) during the 100 ns for three trajectories. D) Visualization of the B‐factor value‐based heatmap of RBDs and NTDs of the representative conformation in the SARS‐CoV‐2 spike trimer simulating systems (left to right: six‐, three‐, and one‐ S416‐bound closed, and apo one‐up open system), blue and red indicated smaller and bigger atomic fluctuation, respectively.

Next, we investigated the synergetic mechanism of the six molecular “bolts” on the stable closed conformation of RBD by decreasing the occupied number of S416 in dynamics simulations. Repeated molecular dynamics simulations of the one‐up apo spike trimer system, closed apo spike trimer system, closed one S416‐bound spike trimer system, closed two S416‐bound spike trimer system, closed three S416‐bound spike trimer system and closed six S416‐bound spike trimer system (3 × 100 ns) using Amber 20 corroborated the persistence of stable interactions between S416 and the spike trimer (**Figure** **3**; Figure S8, Supporting Information). By calculating the root‐mean‐square deviation (RMSD) of the system with respect to the initial structure throughout the simulation, we observed that the RMSD values stabilized after ≈20 ns, signifying that the system had achieved dynamic equilibrium. Consequently, these trajectories were utilized for further analysis. In order to examine variations in the atoms on the protein backbone, the RMSD and the root‐mean‐square‐fluctuation (RMSF) values were calculated relative to the initial structure frame and the average structure of the trajectory, separately. Interestingly, the RMSD of the spike trimer was gradually increased by decreasing the number of bound S416, which indicated that six molecular “bolts” might affect the structural stability of adjacent spike monomer, thus leading to different protein conformations of the whole protein (Figure 3A; Figure S8A, Supporting Information). To more intuitively observe the influence of S416 on the structure of the spike protein, we further compared the average conformation in different systems. It was found that when losing the interactions with the molecular “bolt” S416, the spike structure bolted by them becomes loose, reflected by the larger distances between the RBDs and NTDs core (Figure 3B; Figure S8B, Supporting Information). The core distance between adjacent RBDs and NTDs increased from ≈34 and ≈79 Å to ≈41 and ≈82 Å, respectively (Figure 3B). Meanwhile, the RMSF of the spike RBD domain also gradually increased by decreasing the number of bound S416 (Figure 3C; Figure S8C, Supporting Information), which is reflected by the increasing blue‐to‐red regions of RBDs and NTDs in the B‐factor diagrams (Figure 3D; Figure S8D, Supporting Information). The simulation data revealed that the six molecular “bolts” cooperated with each other to transform the open state of RBD “up” and NTD “out” to the locked state of RBD “down” and NTD “in” of the spike trimer.

### 2.4 Binding Sites at the RBD‐RBD and RBD‐NTD Interfaces are Relatively Conserved in Human‐Infecting Coronaviruses

**Figure 4:**
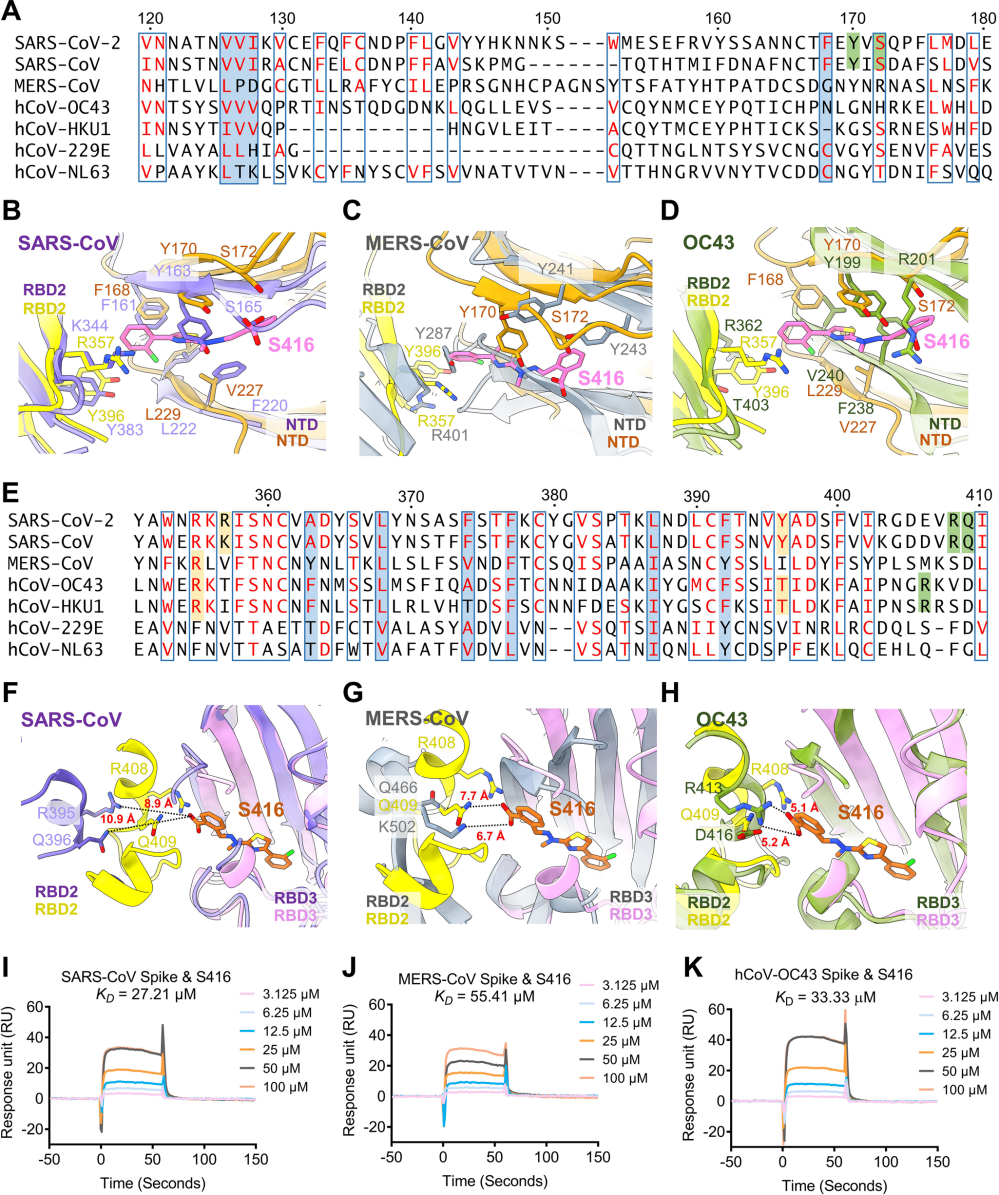
Analysis of human‐infecting coronavirus NTD and RBD architectures reveals relative conservation of the binding pockets at the RBD‐RBD and RBD‐NTD interfaces. A) Sequence alignments of the seven coronaviruses that can infect humans in the NTD‐pocket, and superimposition of NTD (dark goldenrod) of S416‐bound SARS‐CoV‐2 (RBD2 in yellow) with NTDs of ligand free “apo” B) SARS‐CoV (RBD2 and NTD slate blue, PDB ID: 5 × 58), C) MERS‐CoV (RBD2 and NTD slate gray, PDB ID: 5 × 5F), and D) HCoV‐OC43 (RBD2 and NTD olive drab, PDB ID: 6NZK). E) Sequence alignments of the seven coronaviruses that can infect humans in the RBD‐pocket, and superimposition of RBD3 (thistle) of S416‐bound SARS‐CoV‐2 (RBD2 in yellow) with RBDs of ligand free “apo” F) SARS‐CoV (RBD2 and RBD3 slate blue, PDB ID: 5 × 58), G) MERS‐CoV (RBD2 and RBD3 slate gray, PDB ID: 5 × 5F), and H) OC43 (RBD2 and RBD3 olive drab, PDB ID: 6NZK). The conserved residues are highlighted in the composite binding pocket. Residues lining the hydrophobic subpocket are underlaid (light blue). Residues in the adjacent RBD contacting with the S416 hydrophobic tail in the NTD‐pocket are underlaid in orange. Residues positioned to interact with the S416 polar carboxyl group in the NTD‐ or RBD‐pocket are underlaid in green. I–K) SPR analysis of the binding affinities for S416 with the spike protein from SARS‐CoV, MERS‐CoV and HCoV‐OC43.

We further investigated whether the S416 binding pockets at the RBD‐RBD and RBD‐NTD interfaces are conserved in the seven known human‐infecting coronaviruses (SARS‐CoV‐2, SARS‐CoV, MERS‐CoV, HCoV‐OC43, HCoV‐HKU1, HCoV‐229E, and HCoV‐NL63) (**Figure** **4**; Figure S9, Supporting Information). Multiple sequence alignment shows that all the residues lining the hydrophobic subpocket, the two hydrogen‐bonding residues (Y170 and S172) in NTD and the two anchor residues (R357 and Y396) from neighboring RBD in the NTD‐pocket of SARS‐CoV‐2 are fully conserved in SARS‐CoV (Figure 4A; Figure S9A, Supporting Information). Structural alignment of the S416‐bound SARS‐CoV‐2 spike trimer and the apo SARS‐CoV spike trimer (PDB ID: 5 × 58) demonstrates that the S416 binding NTD‐pocket may also present in SARS‐CoV (Figure 4B). Anchor residues Y383 and K344 in the neighboring RBD of SARS‐CoV adopt approximately the same directions as Y396 and R357 in SARS‐CoV‐2, respectively. Without ligand binding, the hydrogen‐bonding residues Y163 and S165 in SARS‐CoV locate lower than Y170 and S172 in SARS‐CoV‐2, making the volume of the NTD‐pocket smaller in SARS‐CoV than that of SARS‐CoV‐2. As for MERS‐CoV, some of the hydrophobic residues lining the subpocket are conserved (Figure 4A; Figure S9A, Supporting Information). The hydrogen‐bonding residue Y170 is conserved with Y241 in MERS‐CoV. Although the two anchor residues (R357 and Y396) from neighboring RBD are not conserved, R401 is aligned well with R357 (Figure 4C), which may serve as the potential anchor residue in the neighboring RBD of MERS‐CoV. However, the S416 binding NTD‐pocket tends to be closed in the apo state of MERS‐CoV due to the upward movement of the antiparallel β sheets. In HCoV‐OC43, the residues lining the hydrophobic subpocket are largely conserved (Figure 4A; Figure S9A, Supporting Information). The hydrogen‐bonding residue Y170 in SARS‐CoV‐2 is aligned well with Y199 in HCoV‐OC43, while S172 is replaced by R201 (Figure 4D), which may be more favorable to form salt bridge interactions with the carboxyl group of S416. In the adjacent RBD, the two anchor residues are not conserved, with R357 replaced by T364 and Y396 replaced by a shorter T403. An arginine is identified around T403 of HCoV‐OC43, which may mimic the role of R357. Like MERS‐CoV, the S416 binding NTD‐pocket between the β sheets of the “apo” HCoV‐OC43 is also in a closed state. Although several residues lining the hydrophobic subpocket are conserved in HCoV‐HKU1 (Figure 4A; Figure S9A, Supporting Information), the hydrogen‐bonding residues Y170 and S172 in NTD and anchor residues R357 and Y396 in the neighboring RBD are not conserved. R187 in the NTD of HCoV‐HKU1^[^ ^11^ ^]^ may influence the shape of the S416 binding NTD‐pocket (Figure S9B, Supporting Information). Because HCoV‐229E^[^ ^12^ ^]^ and HCoV‐NL63 ^[^ ^13^ ^]^ have different folds in NTDs, it is hard to analyze the presence of the S416 binding NTD‐pocket.

Most of the hydrophobic residues lining the subpocket of the S416 binding RBD‐pocket in SARS‐CoV‐2 are conserved in the other six coronaviruses, especially in SARS‐CoV, MERS‐CoV, HCoV‐OC43 and HCoV‐HKU1 (Figure 4E; Figure S9A, Supporting Information). Structural alignments reveal that there is a hydrophobic cavity present in RBDs of SARS‐CoV, MERS‐CoV, HCoV‐OC43 and HCoV‐HKU1 that could be superimposed well with the S416 binding RBD pocket (Figure 4F–H; Figure S9, Supporting Information). The anchor residues R408 and Q409 in the adjacent RBD of SARS‐CoV‐2 are also conserved in SARS‐CoV (R395 and Q396). But R395 and Q396 in the ligand free apo state of SARS‐CoV are located 8.9 and 10.9 Å from the carboxyl group of S416 (Figure 4F). A large shift of the positions of R408 and Q409 could also be observed in the apo SARS‐CoV‐2 (Figure 2F). Although R408 and Q409 are not conserved in the neighboring RBD within MERS‐CoV, two residues K502 and Q466 in the apo structure of MERS‐CoV locating 6.7 and 7.7 Å from the carboxyl group of S416 are identified (Figure 4G), suggesting their potential roles as anchor residues. In HCoV‐OC43, the anchor residues R408 and Q409 are not conserved, but an Arginine (R413) is present within ≈5 Å of the carboxyl group of S416 (Figure 4H). The same situation is also observed in the apo structure of HCoV‐HKU1, where R397 is identified at ≈10 Å from the entrance (Figure S9C, Supporting Information). HCoV‐229E and HCoV‐NL63 have different folds in RBDs,^[^ ^12^, ^13^ ^]^ making it challenging to analyze the presence of the RBD‐pocket.

Subsequently, we determined the binding affinity of S416 for the spike proteins of the six human‐infecting coronaviruses. The results showed that S416 exhibits binding affinities within the *K*_D_ range of 27.21 to 74.69 µm for these viruses (Figure 4I–K; Figure S9D–F, Supporting Information). These findings are in concordance with our conclusions derived from sequential and structural analyses, highlighting the relative conservation of the pockets at the RBD‐RBD and RBD‐NTD‐interfaces among these human‐infecting coronaviruses.

### 2.5 The Molecular “bolt” Inhibits SARS‐CoV‐2 Infection and SARS‐CoV‐2 Spike‐Induced Cell‐Cell Fusion in Human Bronchial Epithelial Cells

**Figure 5:**
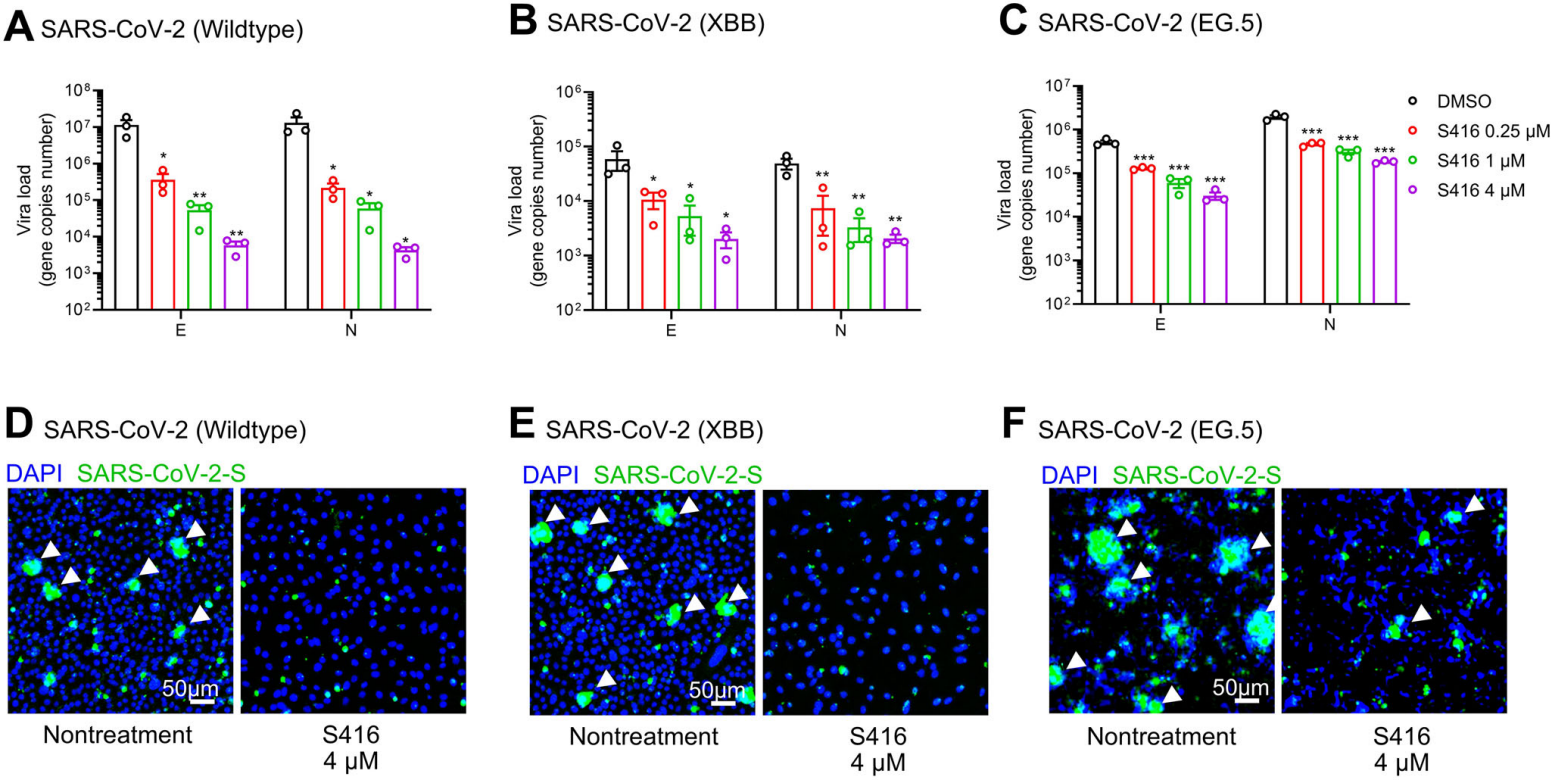
The molecular “bolt” inhibits SARS‐CoV‐2 infection and SARS‐CoV‐2 spike‐induced cell‐cell fusion in human bronchial epithelial cells. A–C) The inhibitory effect of S416 on SARS‐CoV‐2 infection. HBE‐135 E6E7 cells were infected with SARS‐CoV‐2 wildtype (A) Omicron subvariants XBB (B) and EG.5 (C) at a MOI of 3 for 2 h at 37 °C. S416 treatment was initiated at 0 h.p.i., and S416 remained present until 48 h.p.i. when supernatants were harvested to determine viral load by RT‐qPCR targeting the viral E and N genes. D–F) Immunofluorescence assay of cells infected with SARS‐CoV‐2 wildtype (D) XBB (E) and EG.5 (F). HBE‐135 E6E7 cells were infected with SARS‐CoV‐2 following the same procedure as in (A‐C). Cells were fixed and permeabilized for staining with an anti‐viral spike protein antibody, followed by staining with Alexa 488‐labeled secondary antibody. Green represents infected cells. Nuclei were stained with DAPI, and the merge of spike protein and nuclei was shown. Scale *bar*, 50 µm. All data are presented as mean ± SEM from triplicate measurements. Statistical analysis was using one‐way ANOVA for each experiment (*P < 0.05, **P < 0.01, and ***P < 0.001).

To evaluate the antiviral activity of S416 against SARS‐CoV‐2 in the human cell system, we infected human bronchial epithelial cells (HBE‐135 E6E7 cells) with SARS‐CoV‐2 wild‐type and Omicron subvariants (XBB and EG.5 at a MOI of 3). The cells were treated with increasing concentrations of S416. At 48 h post‐infection (h.p.i.), the viral load in cell supernatant was measured by qRT‐PCR targeting the viral E and N genes. As shown in **Figure** **5**A, S416 treatment significantly inhibited the viral load of wild‐type SARS‐CoV‐2 in a dose‐dependent manner, with a reduction of 0.7‐log to 3.0‐log compared to the nontreatment group. Similarly, S416 treatment also reduced the viral load of the SARS‐CoV‐2 Omicron subvariants XBB (Figure 5B) and EG.5 (Figure 5C).

SARS‐CoV‐2 infection typically leads to cell‐cell fusion mediated by spike and ACE2 interaction. We detected the cell‐cell fusion in SARS‐CoV‐2 infected HBE‐135 E6E7 cells by immunofluorescence. In the nontreatment group, wild‐type SARS‐CoV‐2‐infected cells showed intensive cell‐cell fusion (Figure 5D, nontreatment), while infected cells treated with 4 µM S416 not only inhibit virus infection but also inhibit virus transmission via cell‐cell fusion (Figure 5D, S416 4 µm). A similar pattern was observed in HBE‐135 E6E7 cells infected with the Omicron subvariants XBB and EG.5 (Figure 5E,F). These results indicated that S416 could inhibit both SARS‐CoV‐2 infection and spike‐induced cell‐cell fusion in human bronchial epithelial cells.

## 3 Discussion

The binding of the spike to ACE2 is the first step in SARS‐CoV‐2 infection. Therefore, many entry inhibitors, including neutralizing antibodies, recombinant human soluble ACE2, minoproteins, peptides and small molecules, have been discovered to directly or indirectly target Spike, ACE2 and/or their interactions.^[^ ^14^ ^]^ However, the protein‐ and peptide‐ based entry inhibitors, with some being approved in the market or tested in late stages of clinical trials, advance further than the small molecular entry inhibitors in the drug development pipeline.^[^ ^14^, ^15^ ^]^ In comparison, small molecular genome replication inhibitors targeting viral RdRp and 3CL protease have achieved clinical success.^[^ ^15b^ ^]^ Therefore, much effort is needed to develop small molecular entry inhibitors. In screening entry inhibitors against our in‐house compound library, we were thrilled to find this amphipathic molecule S416 could inhibit SARS‐CoV‐2 spike‐mediated cellular entry by blocking the recognition of S/ACE2. S416 does not directly bind to the S/ACE2 interaction interface but instead occupies specific RBD‐RBD and RBD‐NTD interfaces within the spike trimer, thereby influencing the spike's conformational dynamics and promoting its transition to the ACE2 inaccessible state. According to the literature, we report the first Cryo‐EM structure of the all RBD “down” spike trimer locked by six identical small molecules, providing a profound structural basis and novel binding mechanism for developing spike‐targeted entry inhibitors.

**Figure 6:**
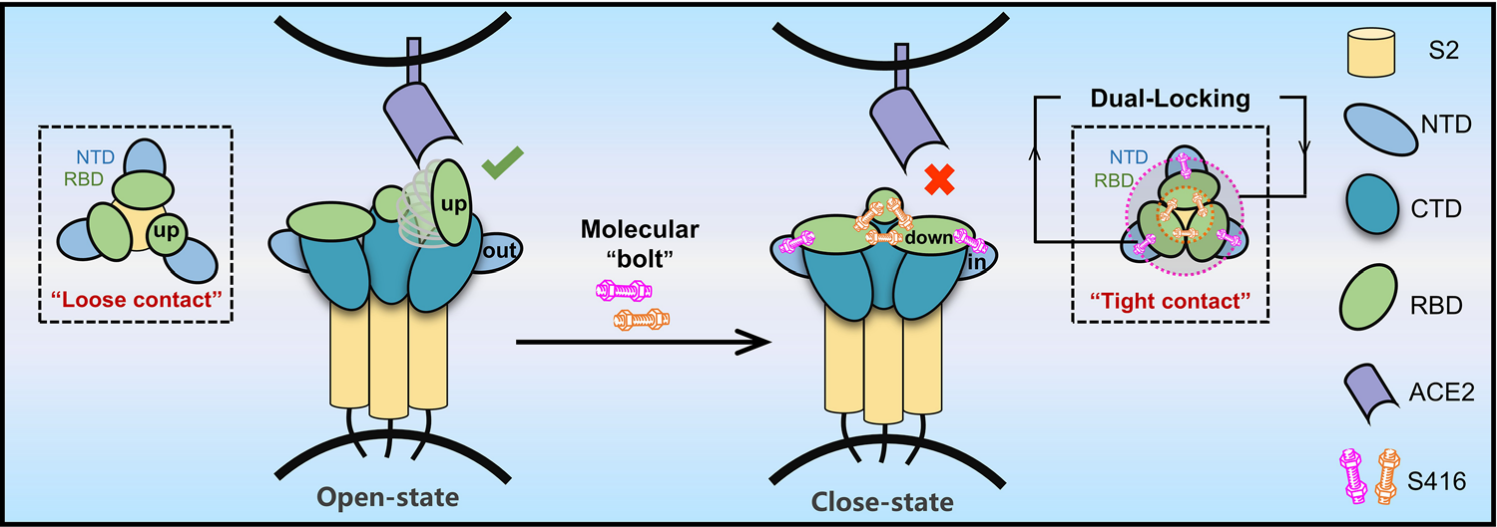
The molecular “bolts” mechanism is proposed for the conformational transition between open and locked states of SARS‐CoV‐2 spike trimer at the RBD‐RBD and RBD‐NTD interfaces. The trio of molecular “bolts” at each RBD‐RBD interface, complemented by another trio at the RBD‐NTD interfaces, effectively dual‐locking the interaction between adjacent monomers of the spike protein. This binding mechanism robustly stabilizes the spike trimer at a rigid and fully locked conformation, serving as novel potential broad‐spectrum drug binding sites for emerging coronaviruses.

The SARS‐CoV‐2 spike protein is a highly mutable and essential target for antiviral interventions.^[^ ^3b^ ^]^ The dynamic nature of the RBD presents challenges for the design of drugs and vaccines that can withstand the pressure of viral mutations.^[^ ^16^ ^]^ Furthermore, the potential of the NTD has not been fully realized in therapeutic strategies.^[^ ^17^ ^]^ Our research introduces a novel approach to stabilizing the close state of the spike protein, achieved by targeting both the RBD‐RBD and RBD‐NTD interfaces. The binding pockets at the RBD‐RBD and RBD‐NTD interfaces share similar hydrophobic and electrostatic properties. As an amphipathic molecule, S416 has a polar carboxyl head and a nonpolar phenylthiazole tail to complement these interfaces. Each bound S416 molecule acts as a “bolt” and cooperates with each other to tighten the inter‐domain contacts of “down” RBD‐RBD and RBD‐NTD. This dual‐locking mechanism, illustrated in **Figure** **6**, results in a stabilized closed conformation of the spike trimer, as evidenced by the absence of RBD “up” states in our cryo‐EM studies. Consistent with this stabilization, Cryo‐EM analysis revealed stronger electron density for glycans, particularly on the S1 domain, in the S416‐bound spike trimer compared to the apo form (data not shown), suggesting that the more rigid trimer conformation enhances glycan visualization. Supporting these structural findings, our molecular dynamics simulations demonstrated reduced overall dynamics and atomic fluctuations in the spike trimer when it is double‐locked at the intermonomeric RBD‐RBD and RBD‐NTD interfaces. This enhanced stability is expected to provide a more substantial barrier to the conformational changes required for viral entry, thus representing a promising therapeutic strategy. For the current MD simulations, our primary objective was to isolate the direct influence of S416 binding on spike protein conformational dynamics; therefore, we chose to exclude glycans to avoid potential confounding effects from their complex interactions. However, we recognize the significant impact of glycans on spike protein behavior,^[^ ^18^ ^]^ and our future investigations will employ enhanced sampling methods, such as aMD,^[^ ^19^ ^]^ to further elucidate the mechanistic impact of S416 binding on spike protein dynamics in the presence of glycans.

S416 is a novel compound with thiazole scaffold designed and synthesized by our group. Originating from our thiazole compound library, which was initially screened and identified as a source of potential dihydroorotate dehydrogenase (DHODH) inhibitors, S416 has demonstrated potent DHODH inhibition, effectively disrupting *de novo* pyrimidine biosynthesis.^[^ ^20^ ^]^ Furthermore, S416 exhibited broad‐reactive antiviral capability.^[^ ^21^ ^]^ Intriguingly, our current investigation reveals a novel mechanism of action for S416: it functions as a molecular “bolt” by binding to the spike trimer interfaces, indicating a structural similarity between druggable pockets in DHODH and the spike trimer, enabling synchronous targeting by S416. We believe that other amphipathic molecules with similar polar‐nonpolar duality could also exhibit comparable binding behavior.

In the study of Toelzer et al.,^[^ ^22^ ^]^ they discovered that a free fatty acid‐ linoleic acid (LA) can bind the RBD‐RBD interface to stabilize the spike trimer in a locked conformation. Intriguingly, our study independently identified an identical RBD‐RBD interface as a binding site for S416, a compound with a distinct chemical scaffold. This convergent finding further validates the RBD‐RBD interface as a highly druggable pocket. Moreover, in contrast to LA, S416 exhibits a unique dual‐locking mechanism by concurrently targeting an additional RBD‐NTD interface, which is not reported before. Such properties enable S416 to have broader spike binding capacity, extending to coronaviruses such as HCoV‐HKU1 (Figure S9, Supporting Information), which was not targeted by LA in Toelzer's study. According to the sequential and structural analysis of human‐infecting coronavirus NTD and RBD architectures, the critical residues of the S416 bound RBD‐RBD and RBD‐NTD interfaces in SARS‐CoV‐2 are fully conserved in SARS‐CoV, largely conserved in MERS‐CoV, and partially conserved in commonly circulating HCoV‐OC43 and HCoV‐HKU1. The remaining two common coronaviruses HCoV‐229E and HCoV‐NL63 have limited conservation. The results of the SPR assay align with the conclusions drawn from our sequential and structural analyses, underscoring the notable conservation of both the NTD‐pocket and RBD‐pocket across human‐infecting coronaviruses. This conservation is promising for the development of broad‐spectrum antivirals, as it implies that our “dual‐ locking” strategy could be effective against a variety of coronavirus strains.

The significant inhibitory effect of S416 on SARS‐CoV‐2 infection (both wild‐type and Omicron subvariants, including XBB and EG.5) and spike‐induced cell‐cell fusion in human bronchial epithelial cells is a pivotal result. It confirms the potential of our strategy to prevent viral entry and spread. The discovery of S416 and its mechanism of action opens new avenues for drug development against COVID‐19 and potentially other coronaviruses. By stabilizing the spike protein in a non‐infectious state, our approach could lead to prophylactic or therapeutic agents that prevent or curtail viral spread. TMPRSS2^[^ ^8h^ ^]^ and cathepsins^[^ ^23^ ^]^ are host cellular factors that facilitate viral entry and therefore represent host‐directed antiviral targets. In contrast, our spike trimer‐targeting agent acts directly on the virus, inhibiting its function prior to host cell entry. Consequently, a promising therapeutic strategy for future investigation is the combination of host‐targeted antivirals (HTAs), such as TMPRSS2 and cathepsin inhibitors, with direct‐acting antivirals (DAAs) like spike trimer lockers. This dual approach could offer synergistic antiviral effects by simultaneously disrupting host cell dependency and directly neutralizing the virus.

## 4 Experimental Section

### NanoBiT‐Based SARS‐CoV‐2 S‐RBD/ACE2 Interaction Assays

The recombinant plasmids of SARS‐CoV‐2 S‐RBD and ACE2 fusion proteins were constructed according to a previously published method.^[^ ^24^ ^]^ Briefly, the NanoBiT PPI Vectors (CS1603B32, Promega, Madison, WI) were used to acquire the fusion expression plasmids of SARS‐CoV‐2 S‐RBD (S residues 319 to 591) or ACE2 (residues 19 to 615) with NanoBiT LgBiT or SmBiT subunit of NanoLuc luciferase according to the manufacturer's instructions. The expression vector encoding fusion protein (HaloTag‐SmBit) was served as a negative control.

The initial screening and IC_50_ determination of SARS‐CoV‐2 S‐RBD/ACE2 interaction inhibitors were performed as previously described.^[^ ^24^ ^]^ Briefly, HEK293 cells were seeded into a 6‐well cell culture plate and transiently co‐transfected with SARS‐CoV‐2 S‐RBD‐LgBiT and SmBiT‐ACE2 fusion plasmids using FuGENE HD transfection reagent (Promega, Madison, WI). After 6 h, HEK293 cells were reseeded into a 384‐well plate for 16–24 h and the test compounds were added. Following 3 h of incubation, Nano‐Glo live Cell Assay reagent was added, and luminescence was determined using the Envision plate reader (EnVision, Perkin Elmer, Waltham, MA, USA). To exclude the false positives, the cell viability of the compounds on HEK293 cells was carried out according to the same transfection conditions using the CellTiter‐Glo Luminescent Cell Viability Assay (Promega, Madison, WI). At the same time, the inhibitory effects of the compounds on NanoLuc (HEK293/Nanoluc stable cells) were also measured with Nano‐Glo live Cell Assay reagent. The activities of the compounds were evaluated in a dose‐dependent manner to analyze the inhibitory effects (IC_50_) on SARS‐CoV‐2 S‐RBD/ACE2 interaction (NanoBiT inh%), NanoLuc luciferase (NanoLuc inh%) and the cell proliferation (Cytotox inh%, CC_50_) on HEK293 cells.

### SPR Based Binding Affinity Assays

SPR experiments were conducted using a BIAcore T200 to evaluate the binding affinities of S416 binding to the recombinant SARS‐CoV‐2 S‐trimer protein (Catalog #: DRA49, Novoprotein Technology Co., Ltd) and S‐RBD protein (Catalog #: DRA42, Novoprotein Technology Co., Ltd), HCoV‐NL63 spike protein (Catalog #: 40604‐V08B, Sino Biological), HCoV‐229E spike protein (Catalog #: 40605‐V08B, Sino Biological), HCoV‐OC43 spike protein (Catalog #: 40607‐V08B, Sino Biological), HCoV‐HKU1 S‐trimer protein (Catalog #: DRA140, Novoprotein Technology Co., Ltd), MERS‐CoV S‐trimer protein (Catalog #: DRA95, Novoprotein Technology Co., Ltd), and SARS‐CoV S‐trimer protein (Catalog #: DRA96, Novoprotein Technology Co., Ltd). The proteins SARS‐CoV‐2 S‐trimer was diluted in sodium acetate solution pH 4.0 to a final concentration of 100 µg mL^−1^. Other proteins were diluted in sodium acetate solution pH 4.5 to a final concentration to 50 µg mL^−1^. Then they were immobilized covalently on a CM5 sensor chip with the final immobilization levels equal to 15 154.8 resonance units (RU) (SARS‐CoV‐2 S), 5008RU (S‐RBD), 2000RU (HCoV‐NL63), 6177.5RU (HCoV‐229E), 5000RU (HCoV‐OC43), 6992.3RU (HCoV‐HKU1), 4029.4RU (MERS‐CoV), and 3487.5RU (SARS‐CoV S) respectively. The running buffer was PBS, 0.005% (vol/vol) surfactant P20, pH 7.4, and 1% DMSO. S416 was diluted using the running buffer from the top concentration. The measurements were performed at a flow rate of 30 µL min^−1^. For each binding cycle, the analyte was injected for 90 or 60 s and the dissociation time was 180 or 90 s. Data were analyzed using the BIAevaluation 1.1 software.

### Inhibition of Pseudotyped SARS‐CoV‐2 Infection

The inhibitory activity of S416 against the infection of pseudotyped SARS‐CoV‐2 was evaluated. 293T cells were transiently transfected with three plasmids which were pcDNA3‐SARS‐CoV‐2 S, pAX2 and pHB‐Rluc (encoding for Luciferase gene), respectively. SARS‐CoV‐2 Spike protein was used as envelope protein, and luciferase gene was packaged inside the virus. Pseudotyped particles were efficiently released in the supernatant. The supernatant was harvested at 48 h post‐transfection. 293T cells transiently transfected with the pcDNA3.1‐ACE2 plasmid were used as the target cells. Target cells were plated in a 96‐well plate for 12 h, and then serial dilutions of S416 were added to the target cells to incubate for 1 h. After that, pseudoviruses were added. The medium was changed after 24 h and incubation continued for 48 h. Renilla luciferase reagent was added to each well to record the luminescent signals. The number of pseudoviruses entering the target cells was calculated by detecting luciferase expression.

### 1D NMR Spectroscopy Experiment

Carr‐Purcell‐Meiboom‐Gill (CPMG) and saturation transfer difference (STD) experiments were carried out at 25 °C on a Bruker Avance III‐600 MHz (proton frequency) spectrometer equipped with a cryogenically cooled probe (Bruker biospin). NMR buffer was 1 x PBS (dissolved in D2O), 5% deuterated dimethyl sulfoxide (DMSO‐d6). Samples containing 200 µm S416 (dissolved in NMR buffer) in the absence or presence of 5 µm SARS‐CoV‐2 RBD were used in NMR spectroscopy data acquisition.

### Drug Affinity Responsive Target Stability (DARTS) Assay

S‐RBD protein was diluted to 50 µg µL^−1^. S416 was added and its final molar concentration was 10‐ or 100‐fold of S‐RBD. As a vehicle control, the solvent was added to S‐RBD protein. After incubation for 30 min, S‐RBD protein was digested by adding pronases. The mass ratio between pronase and RBD was 1:30, 1:40, 1:50 or 1:60. The reaction was stopped after 30 min. Each sample was assayed by Coomassie brilliant blue staining and silver staining of the SDS‐PAGE gel. For every sample in one piece of gel, the total amount of initial protein in the samples used for electrophoretic analysis was consistent.

### Dynamic Light Scattering (DLS)

S416 were prepared in filtered 50 mm KPi buffer, pH 7.0 with final DMSO concentration at 1% (vol/vol). Colloidal particle formation was detected using Zetasizer Nano ZS3600 (Malvern Panalytical Ltd). The compound was screened at 100 µM, eight‐point half‐log dilutions of S416 was performed in triplicate.

### Enzyme Inhibition Assays

S416 were prepared in 50 mm KPi buffer with a final DMSO concentration of 1% (vol/vol) as described previously. Different concentrations of S416 were incubated with 2 nm AmpC β‐lactamase (AmpC) (Catalog #: S24646, Shanghai yuanye Bio‐Technology Co., Ltd) or Malate dehydrogenase (MDH) (Catalog #: S10168, Shanghai yuanye Bio‐Technology Co., Ltd) for 5 min. The AmpC reaction was initiated by the addition of 100 µm CENTA chromogenic substrate (Catalog #: E152504, Shanghai EFE Bio‐Technology Co., Ltd), and the absorbance was rapidly detected at 405 nm for 80 s. The MDH reaction was initiated by the addition of 200 µm nicotinamide adenine dinucleotide (NADH) and 200 µm oxalylacetate, and the absorbance was rapidly detected at 340 nm for 80 s. Initial rates were divided by the DMSO control rate to determine percent enzyme activity (%).

### Cryo‐EM—Expression and Purification of the Spike Protein

The recombinant spike protein (S‐ECD protein, 14–1208 residues, GenBank: MN908947) was cloned into the pFastbac1 vector (Gibco) with two proline substitutions at residues 986 and 987, and a “GSAS” substitution at residues 682 to 685. The protein contained an N‐terminal GP67 signal peptide and C‐terminal followed by His tag. The constructed pFastbac1 vector was transformed into DH10Bac to generate recombinant bacmid. The recombinant baculovirus was amplified in Sf9 cells at a density of 2 × 10^6^ per ml with the Bac‐to‐Bac system. Sf9 insect cells were infected with baculovirus at a ratio of 1:30 when the cell density reached 4 × 10^6^ per ml. After 60 h, the Sf9 cell supernatants were harvested and transferred to a large beaker. A heavy white precipitate formed immediately when the supernatant was added with 50 mm Tris (pH 8), 1 mm NiCl_2_, and 5 mm CaCl_2_.The suspension was stirred for at least 45 min at room temperature. After spinning at 4000 rpm,10 min at room temperature, the supernatants were collected and loaded with 2 ml nickel resin per 1 L supernatant for 1 h in a cold room. Then, the nickel resin was collected and washed with 100 ml wash buffer containing 20 mm HEPPES (pH 7.5), 500 mm NaCl and 20 mm imidazole. At last, the spike protein was eluted with 20 mm HEPES (pH 7.5), 100 mm NaCl and 250 mm imidazole. The eluted protein was concentrated in an ultrafiltration tube (Sigma‐Aldrich, Amicon Ultra‐15 ml) and further purified with Superose 6 Increase 10/300 GL (GE Healthcare Life Sciences). The peak fraction was concentrated with an ultrafiltration tube and fast frozen in liquid nitrogen until use.

### Cryo‐EM Grid Preparation and Data Collection

For cryo‐EM grid preparation, the Au 300 grids (Quantifoil R1.2/1.3) were pre‐discharged with Pie Scientific Tergeo Plasma Cleaner. The purified spike protein was concentrated to 3 mg ml^−1^ and loaded on the grids. The grids were blotted for 4 s at 10 °C and 100% humidity and flash‐frozen in liquid ethane using Vitrobot (Mark IV, Thermo Fisher Scientific).

For data collection, the grids were checked and the cryo‐EM data were collected on 300 kv Titan Krios Gi3, which was equipped with a GIF‐quantum energy filter (Thermo Fisher Scientific, USA) and Gatan K3 BioQuantum camera. All movies were automatically acquired at a magnification of 105000 X with SerialEM 3.8 software. The movie stacks were 50 frames with a 20 ev slit width and the pixel size was 0.83 Å. The defocus range was from ‐1.0 to ‐2.0 µm.

### Cryo‐EM Data Processing

For Apo‐spike protein, 3448 movies were performed by cryoSPARC v4.1.1. Each movie stack was aligned with Patch motion correction. A total number of 2858021 particles were extracted with the auto‐picking. After 3 rounds of 2D classification, the good particles were reduced to 282782. The particles were further reduced their size to 177481 by 3D classification and Ab‐initio reconstruction. The 2.86 Å resolution density map at FSC 0.143 was obtained when the initial map of the particles was performed with Homogeneous Refinement, Non‐uniform Refinement and Local Refinement.

For S416‐spike protein, 7281 movies were performed by cryoSPARC v4.1.1. In the auto‐picking, a total number of 5921528 particles were extracted. After 3 rounds of 2D classification, the good particles were reduced to 382872. Then, the particles were reduced their size to 174780 by 3D classification and Ab‐initio reconstruction. The 2.95 Å resolution density map at FSC 0.143 was obtained when the initial map of the particles was performed with Homogeneous Refinement, Non‐uniform Refinement and Local Refinement.

### Cryo‐EM Model Building

The atomic model (PDB: 6VYB) was used as the initial template to fit the cryo‐EM map of the Apo‐spike protein structure. The S416‐spike protein structure was generated with the model (PDB:7JJI) as a template. All models were subsequently docked into the density maps using the UCSF Chimera and manually refined and rebuild in the COOT 0.9.7 and Phenix.Real_sapce_refinement. The final refinement model statistics were validated by Phenix (Table S1, Supporting Information). The molecular graphics figures were presented using UCSF Chimera, UCSF ChimeraX and PyMOL.

### Molecular Dynamics Simulations

The structures for the one‐up apo spike trimer system and totally closed six S416‐bound spike trimer system were from our Cryo‐EM study. The closed apo spike trimer system, closed one S416‐bound spike trimer system, closed two S416‐bound spike trimer system, closed three S416‐bound spike trimer system, and totally closed six S416‐bound spike trimer system were built by deleting six S416 molecules, five S416 molecules, four S416 molecules, three S416 molecules from the closed six S416‐bound spike trimer system, respectively. Recognizing the significant impact of glycans on spike protein behavior,^[^ ^18^ ^]^ the systems were simplified by removing them to concentrate on core protein dynamics in this study. The above structures were prepared with the Protein Preparation Wizard available in Maestro (Schrodinger, Inc., version 10.2). Before the simulations, the missing residues in the middle of a chain were added using the Prime program in Maestro. Hydrogen atoms were added and minimized with the OPLS‐2005 force field. Then, the protonation states of the ionizable residues were computed by using the PROPKA online server. The three monomers have consistent protonation states, for example, residues H23, H40, H175, H900, and H916 were assigned to be protonated at the ε position, H43 was protonated at both nitrogen atoms. Geometric optimization of the ligands was conducted at the HF/6‐31G* level by using Gaussian09, followed by the calculation of the RESP charges. Ligand parameters were generated with Antechamber with GAFF. MD simulations were performed and analyzed with the Amber 20 package. All atom force field ff14SB was applied. Protein‐ligand complexes were solvated by using the TIP3P water model. Na^+^ ions were added to neutralize the systems. The systems were minimized with descending restraint forces. The temperature was slowly increased from 0 to 300 K under NVT ensemble conditions and was then equilibrated under NPT ensemble conditions for 500 ps at 300 K. Langevin dynamics was adopted with a collision frequency of 1.0 ps^−1^. Ten Angstrom was specified for nonbonded cutoff and the particle mesh Ewald (PME) method was used for long‐range electrostatic interactions. Then, 100 ns unrestrained MD production was performed for each system. The time step was set to 2 fs (the SHAKE algorithm is activated) and the atom coordinates were saved every 20 ps for subsequent analysis. After the simulations, the “CPPTRAJ” was used to analyze the coordinate trajectories, the average conformation is calculated as the merged last 50 ns trajectory of the three replicate simulations using command “average av.pdb * pdb nobox”.

### Domain Rearrangement Analysis

Domain rearrangement analysis was performed using available cryo‐EM structures for SARS‐CoV‐2. Domains for the vector analysis were selected based on visual inspection of alignments between apo and S416‐bound SARS‐CoV‐2 structures. Specifically, Cα centroids were determined for the S1 NTD (residues 27–43 and 54–271), RBD (330–443 and 503–528), SD1 (323–329 and 529–590), and SD2 (294–322 and 591–696), respectively; as well as a β‐sheet motif in the NTD (residues 116–129 and 169–172) and a helix motif in the RBD (residues 403–410). Cα centroids in the S2 subunit were obtained for a β‐sheet motif (residues 717–727 and 1047–1071) and the CD domain (711–716 and 1072–1122). The central axis was calculated by Cα centroids of NTD and RBD. Domain rearrangements between these centroids were determined and used in the subsequent analysis using PyMOL 2.3.3.

### Multiple Sequence Alignment

The sequences of the spike protein from SARS‐CoV‐2 (UniProt ID: P0DTC2), SARS‐CoV (UniProt ID: P59594), MERS‐CoV (UniProt ID: K9N5Q8), HCoV‐OC43 (UniProt ID: P36334), HCoV‐HKU1 (UniProt ID: Q5MQD0), HCoV‐229E (UniProt ID: P15423), and HCoV‐NL63 (UniProt ID: Q6Q1S2) were downloaded from the Universal Protein Resource (https://www.uniprot.org/) in FASTA file format and then imported and analyzed using the MSA (multiple sequence alignment). The results of the alignment and the consensus sequences were visualized using ClustalX.^[^ ^25^ ^]^

### Viruses

The SARS‐CoV‐2 wild‐type and Omicron XBB and EG.5 subvariants were isolated by the Navy Medical University. The virus was multiplied in Vero E6 cells to make virus stock. All experiments involving live SARS‐CoV‐2 were conducted in a Biosafety Level‐3 (BSL‐3) laboratory at the Navy Medical University, following approved standard operating procedures.

### Real‐Time Reverse‐Transcriptase–Polymerase Chain Reaction

The mRNA levels of the indicated genes were quantified via quantitative PCR with reverse transcription (qRT‐PCR). Purified RNAs extracted with TRIzol (Catalog #: 15 596 018, Invitrogen) were subjected to reverse transcription using oligo dT and random primer (Catalog #: RR037A, Takara). The expression levels of SARS‐CoV‐2 viral genome in cells were quantified using the TaqMan RT‐PCR assay kit (Catalog #: 11205ES08, Yeason) and compared to an established external standard (Catalog #: GBW(E)09 1111, SIMT) for absolute quantification of viral copy numbers. All the primers and TaqMan probes used in this study are listed in Table S2 (Supporting Information).

### Virus Infection Assay

The human bronchial epithelial cell lines (HBE135‐E6E7) were bought from ATCC Cells (CRL‐2741). HBE‐135 E6E7 cells were infected with SARS‐CoV‐2 wildtype and Omicron subvariants XBB/ EG.5 (MOI = 3) for 2 h at 37 °C. S416 treatment was initiated at 0 h.p.i., and S416 remained present until 48 h.p.i. when supernatants were harvested to determine viral load by RT‐qPCR targeting the viral E and N genes. To detect viral protein expression in HBE135‐E6E7 cells, cells were fixed with 4% paraformaldehyde and permeabilized with 0.5% Triton X‐100. The cells were then incubated with the primary antibody (a polyclonal antibody against the SARS‐CoV‐2 spike protein; homemade) after blocking, followed by incubation with the secondary antibody (Alexa 488‐labeled goat anti‐mice, Catalog #: ab150113, Abcam).

### Statistical Analysis

Raw cycle threshold (Ct) values of the test samples were converted into viral copy numbers using the standard curve. Data analysis and visualization were conducted using Prism 8.0 (GraphPad Software, USA). Graphs represent mean values ± SEM or SD from three independent replicates (*n* = 3), as indicated in the figure legends. For comparisons involving three or more groups with a single variable, statistical analysis was conducted using one‐way ANOVA (*P < 0.05, **P < 0.01, ***P < 0.001).

### Data Availability

All data were available in the main text or the supplementary materials. Atomic coordinates and cryo‐EM maps of the reported structure were deposited into the Protein Data Bank (PDB) and Electron Microscopy Data Bank (EMD) under the session codes PDB 9LVI and EMD‐63420 for SARS‐CoV‐2 spike, and PDB 9LVS and EMD‐63424 for SARS‐CoV‐2 spike in complex with S416.

## Conflict of Interest

The authors declare no conflict of interest.

## Author Contributions

S.L., F.Y., Y.Z., J.W., and H.P. contributed equally to this work. S.L., K.X., Y.D., and H.L. conceived and codirected the study; S.L. and H.L. designed S416; J.W. and S.L. performed the computational simulation studies and structural data analyses; F.Y., B.Z., Z.Y.Z., and G.C. performed Cryo‐EM related studies and structural analyses; Y.Z., H.P., and L.J. performed in vitro antiviral assay in human bronchial epithelial cells and analyzed data; L.Z., T.Y., H.G., J.H., and J.Y.W. performed the SPR assay, cell‐based pseudovirus entry assay, DLS and enzyme (MDH and AmpC) inhibition assay; L.C. performed the NanoBiT assay; Z.J.Z. and X.Q. led the compound synthesis work; S.L. and H.L. interpreted and reviewed all data; S.L., F.Y., Y.Z., J.W., L.Z., and K.X. wrote the manuscript with help from all authors.

## Supporting information



Supporting Information

## Data Availability

The data that support the findings of this study are available in the supplementary material of this article.
