# Supplementary material for: Dual‐Locking the SARS‐CoV‐2 Spike Trimer: An Amphipathic Molecular “Bolt” Stabilizes Conserved Druggable Interfaces for Coronavirus Inhibition
Source: Adv Sci (Weinh). 2025 Apr 26;12(27):2417534. doi: 10.1002/advs.202417534 (PMC12279193; doi:10.1002/advs.202417534)
Supplement: Supplementary file 1 — Supporting Information [file ADVS-12-2417534-s001.docx]

Supporting Information

**Dual-Locking the SARS-CoV-2 Spike Trimer: An Amphipathic Molecular “Bolt” Stabilizes Conserved Druggable Interfaces for Coronavirus Inhibition**

*Shiliang Li^1,2,3†^, Fang Ye^4,†^, Yucheng Zhe^5,†^, Jie Wang^2,†^, Haoran Peng^6,†^, Lili Zhu^2^, Lili Chen^7^, Tao Yu^1^, Huan Ge^2^, Jiaqi He^2^, Binghao Zhang^4^, Jiayun Wu^2^, Zhiyi Zhang^4^, Liangliang Jiang^6^, Geng Chen^4^, Ping Zhao^6^, Ke Lan^5^, Zhenjiang Zhao^2^, Xuhong Qian^1,2^, Ke Xu^5,*^, Yang Du^4,*^, Honglin Li^1,2,8,*^*

**This supplemental information file includes:**

Figures S1 to S9

Tables S1 to S2


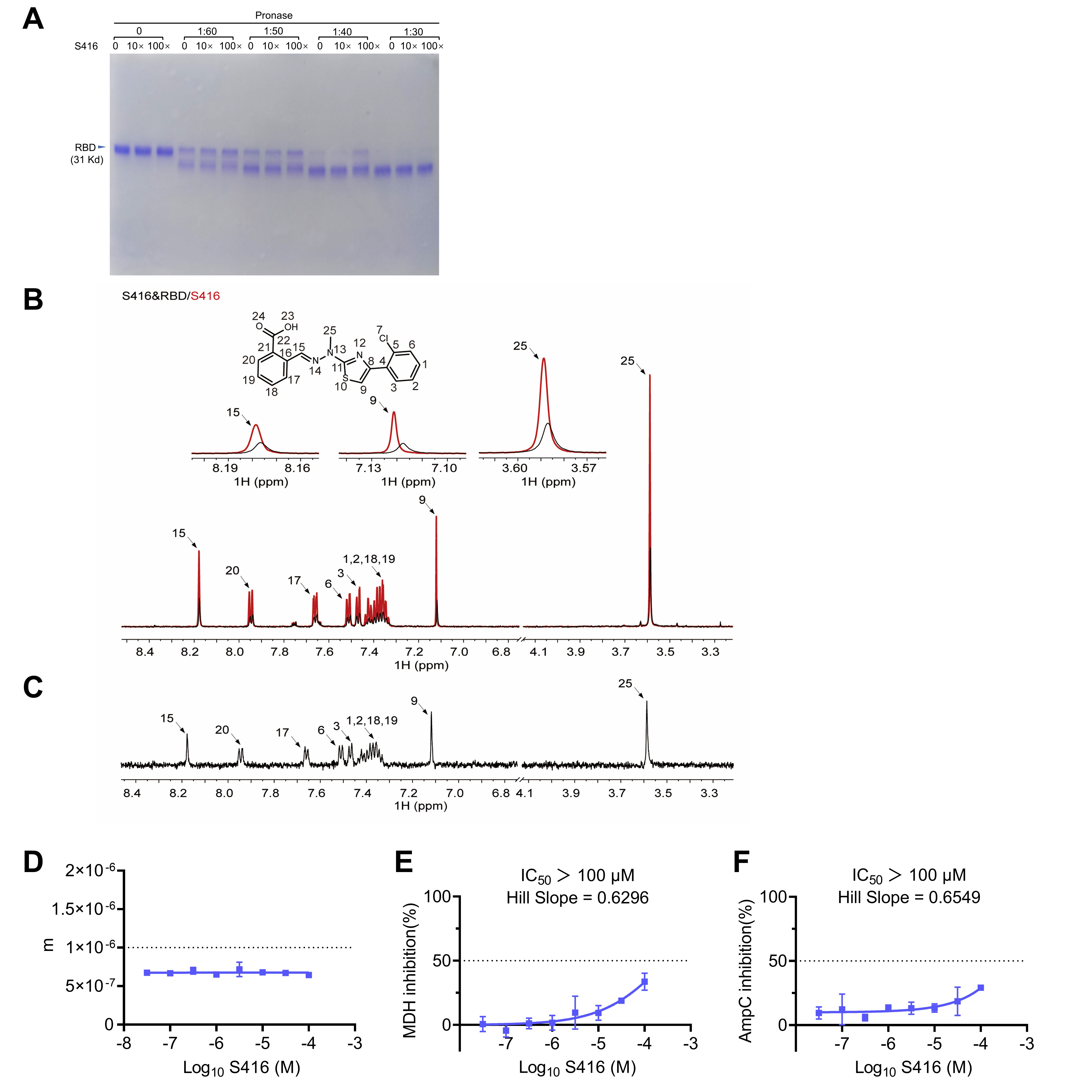


**Figure S1.** **Experimental analysis of the binding of S416 to SARS-CoV-2 S-RBD and the potency of S416 in inhibiting cellular entry.** (**A**) DARTS assay of S416 binding to SARS-CoV-2 S-RBD. (**B**) Ligand observed 1H NMR experiment implicates the binding of S416 to SARS-CoV-2 S-RBD. T1ρ spectra were acquired by using S416 (200 μM) alone (colored in red), and with 5 μM SARS-CoV-2 S-RBD protein (colored in black). (**C**) Saturation transfer difference spectrum recorded for 200 μM S416 in the presence of SARS-CoV-2 S-RBD (5 μM). NMR data for protons in the structure of S416 were generally assigned. (**D**) via dynamic light scattering (DLS), along with its inhibition of the counter-screening enzymes MDH (**E**) and AmpC (**F**), demonstrates that S416 does not exhibit colloidal properties in vitro^26^. All measurements are greater than or equal to triplicate.


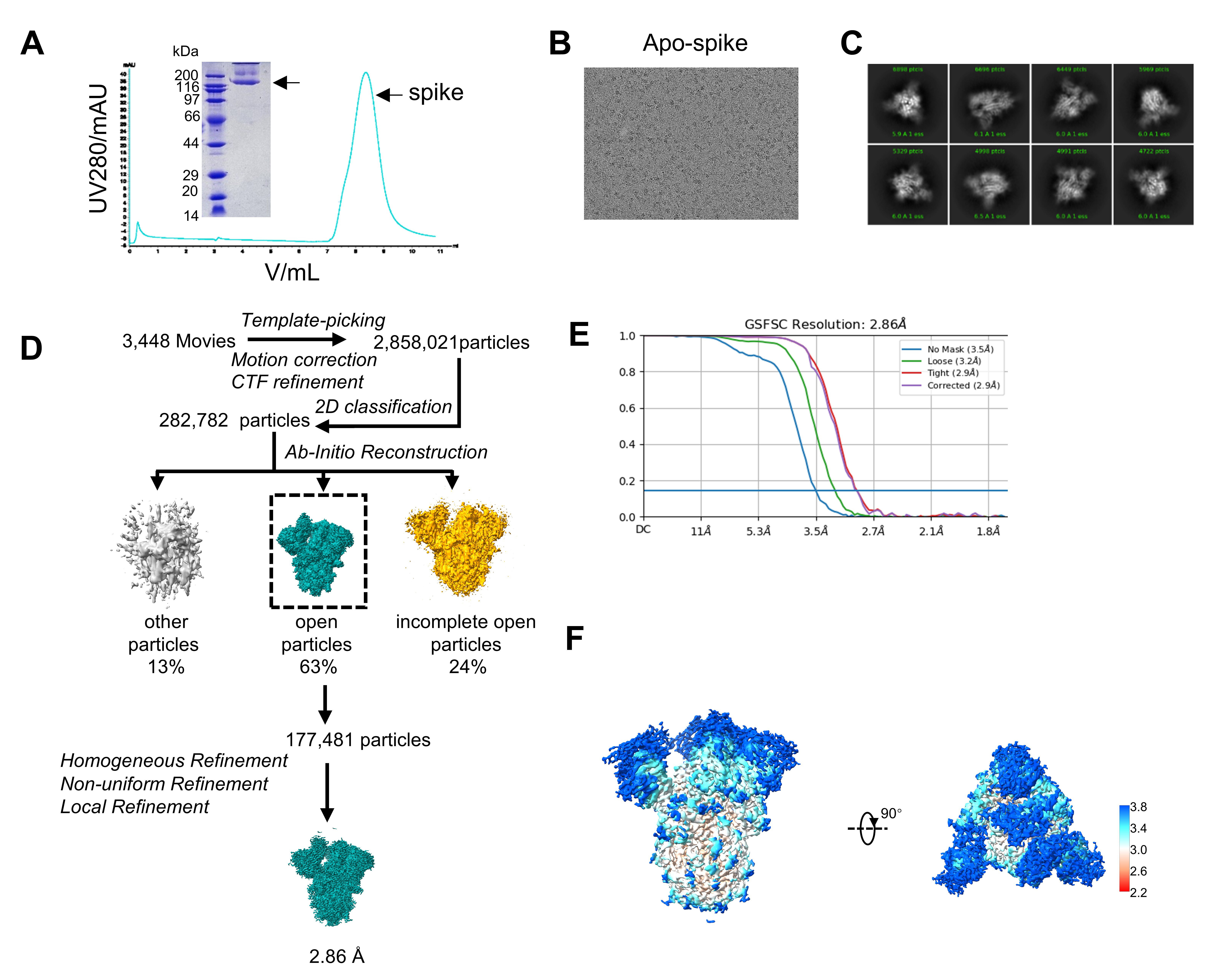


**Figure S2.** **Cryo-EM data and map quality for Apo-spike**. (**A**) SDS-PAGE analysis and size-exclusion chromatogram of the affinity-purified spike-trimer protein. (**B**) Representative micrograph of the spike trimer-DMSO. (**C**) Representative 2D classification result. (**D**) Cryo-EM image processing workflow of the spike trimer-DMSO. The methods section and supplement table provided the details. (**E**) FSC curves for the spike trimer-DMSO. (**F**) Cryo-EM density of the spike trimer-DMSO was shown and colored according to local resolution.


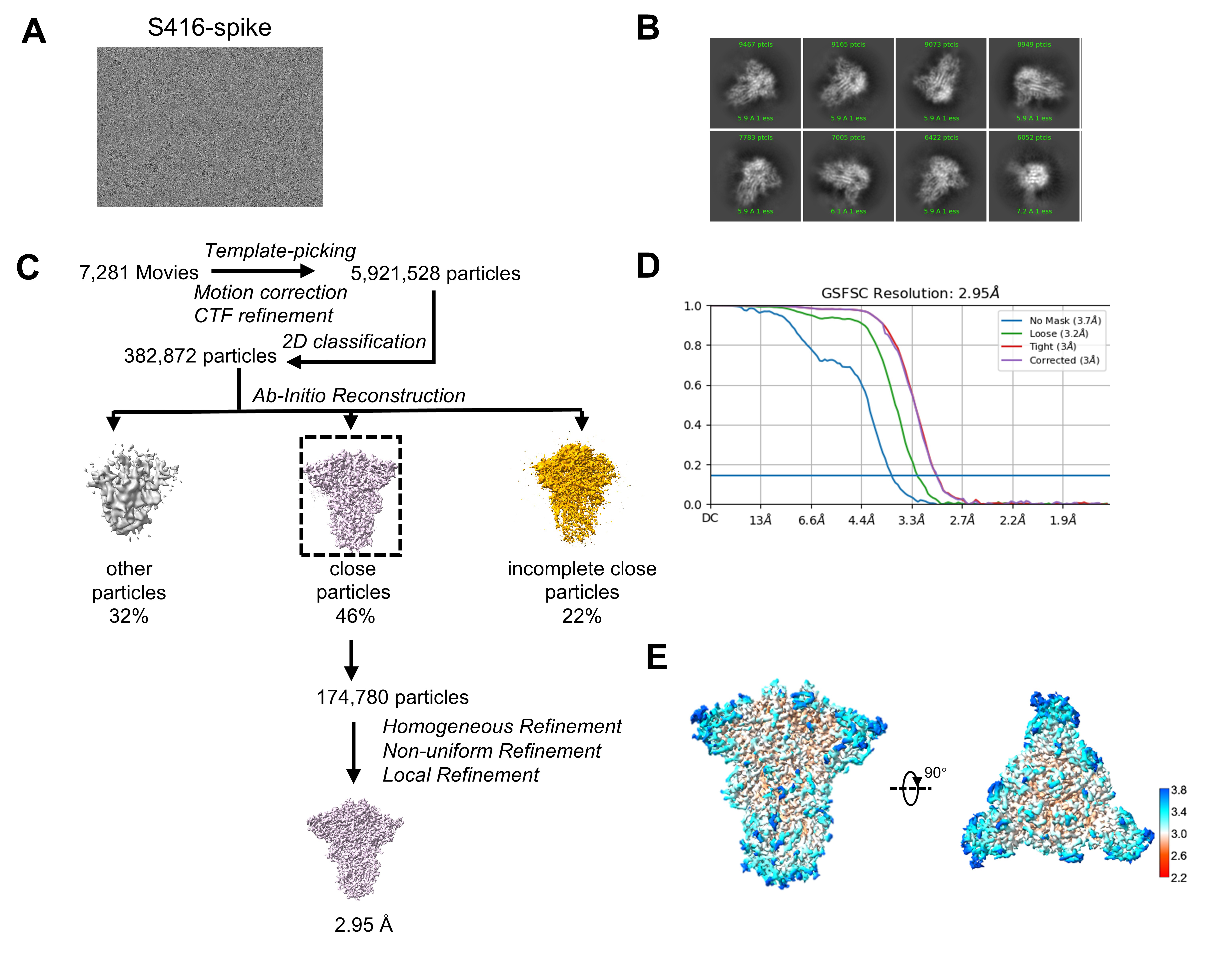


**Figure S3**. **Cryo-EM data and map quality for S416-spike**. (**A**) Representative micrograph of the spike trimer-S416. (**B**) Representative 2D classification result. (**C**) Cryo-EM image processing workflow of the spike trimer-S416. The methods section and supplement table provided the details. (**D**) FSC curves for the spike trimer-S416. (**E**) Cryo-EM density of the spike trimer-S416 was shown and colored according to local resolution.


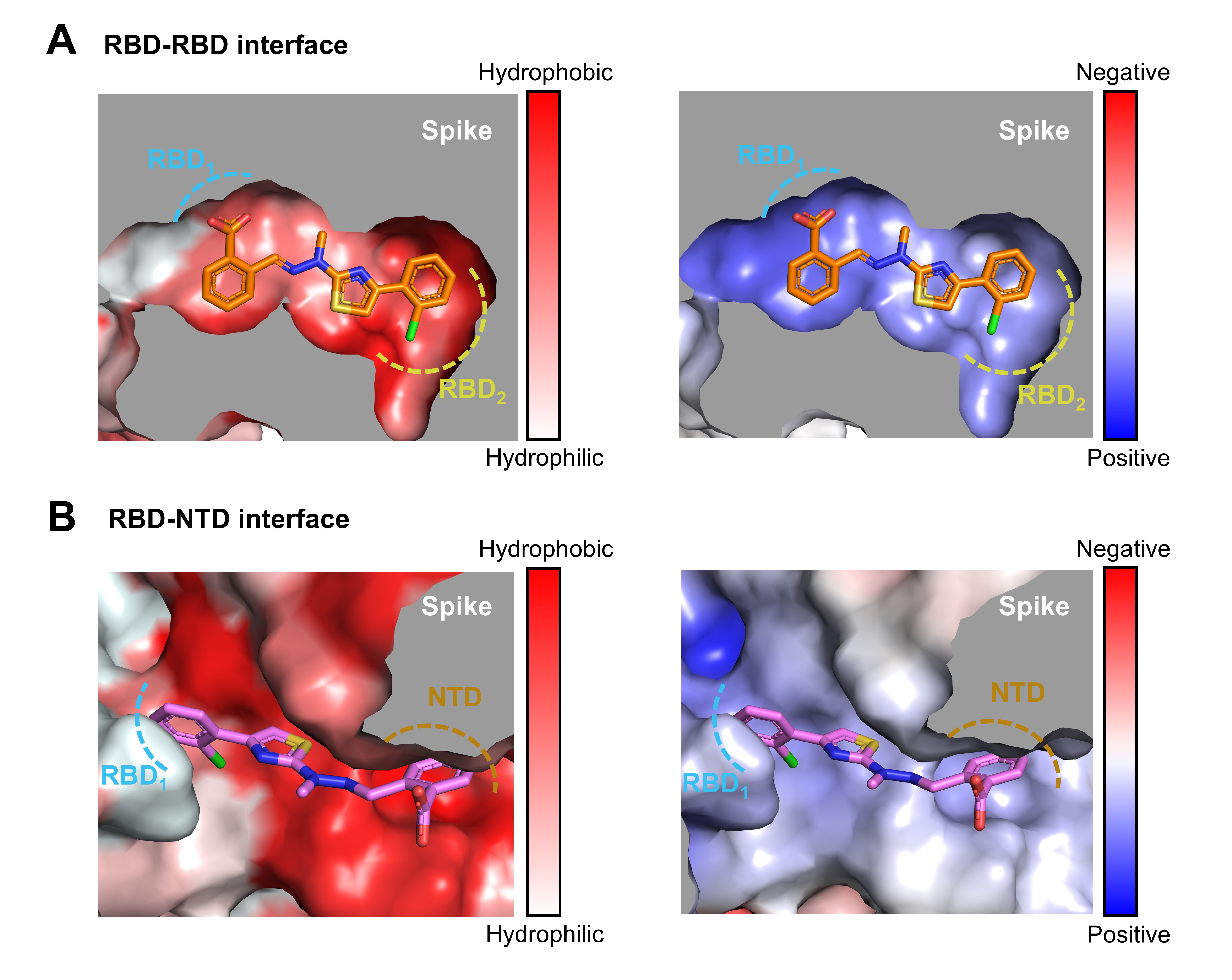


**Figure S4. S416-bound RBD-RBD interface (A) and RBD-NTD interface (B) share similar hydrophobic and electrostatic properties.** The hydrophobic and electrostatic properties of the binding sites were calculated separately by “run_h.py” script and APBS electrostatics plugin in PyMOL 2.3.3.


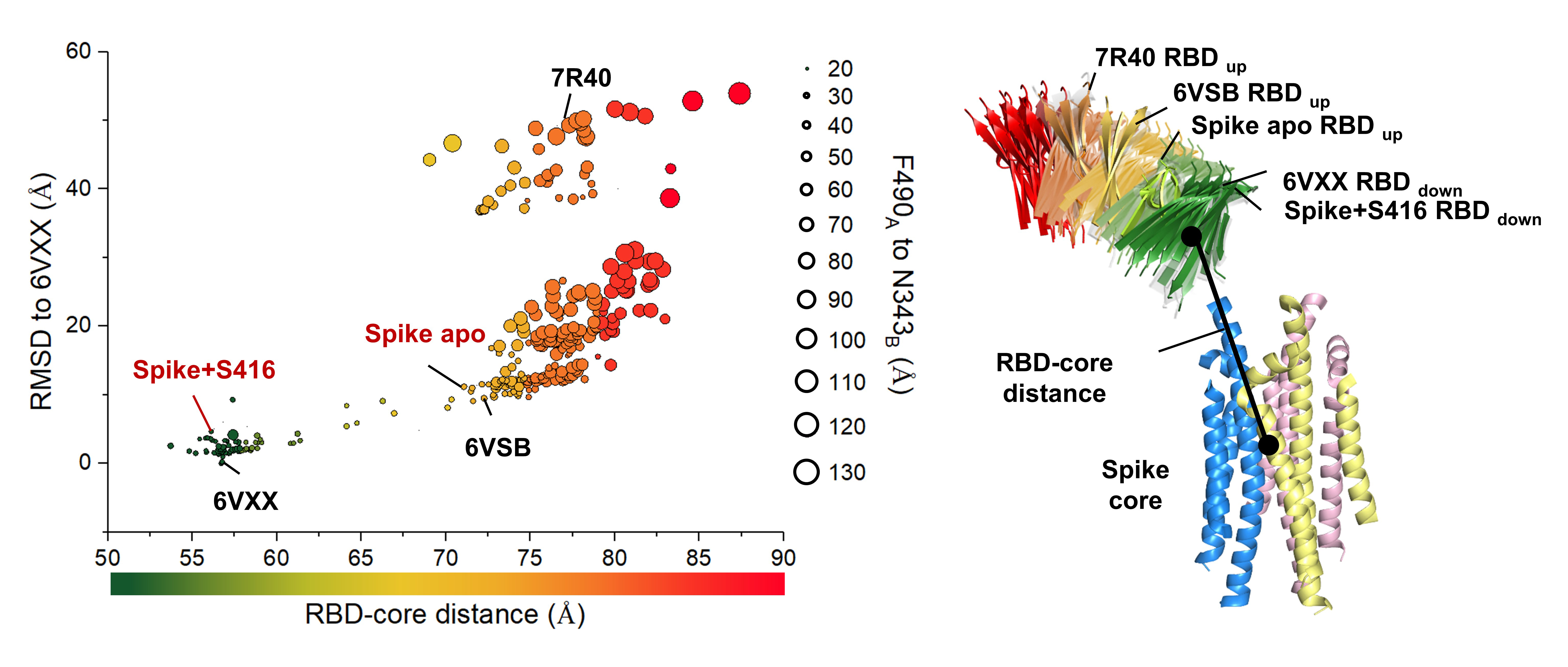


**Figure S5.** **Conformational space analysis of the reported spike structures.** The data plot was calculated based on the RBD's Cα-root-mean-square deviation (RMSD) from our cryo-EM structure and other published spike structures compared to the RBD down state (PDB ID: 6VXX). Data points are colored on the basis of the RBD–core distance and the bubble size depends on the distance between Phe490 and Asn343. The locations of the representative structures are labeled. RBD–core distance was calculated by primary regions of the spike defined for tracking progress of the opening transition.


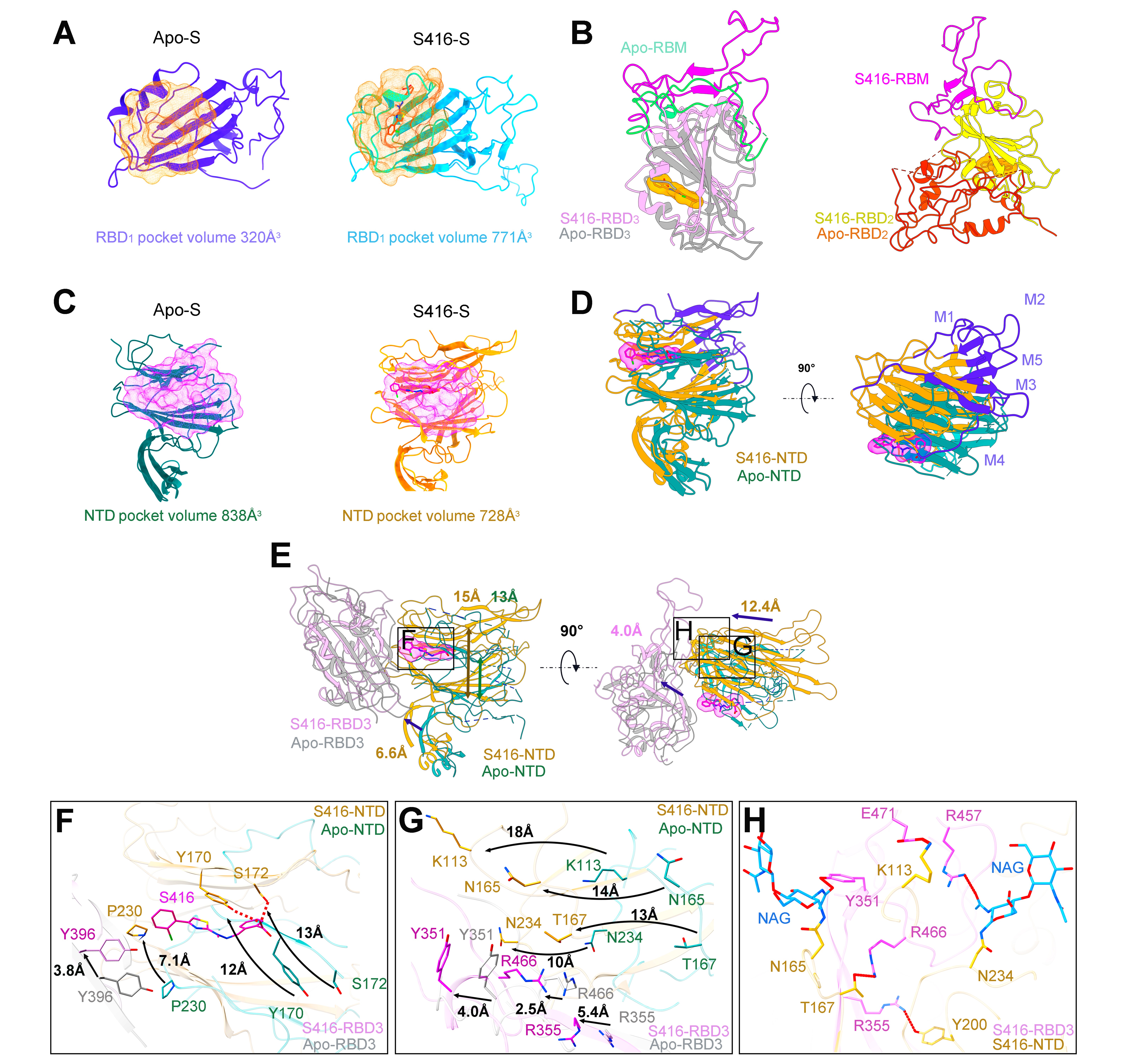


**Figure S6. Comparison of S416-bound and Apo spike protein structures focusing on RBDs and NTDs.** (**A**) Comparison of Apo-spike (medium purple) and S416-spike RBD (deep sky blue) pocket volume. (**B**) Top view of S416-RBD3 (thistle), Apo-RBD3 (dark grey), S416-RBD2 (yellow) and Apo-RBD2 (coral). The motif responsible for ACE2 binding (RBM) of S416-S is in magenta and the RBM of Apo-S is in lime. S416 is shown as orange surface. (**C**) Comparison of Apo-spike (dark cyan) and S416-spike NTD (dark goldenrod) pocket volume. (**D**) Top and side view of Apo-spike (dark cyan) and S416-spike NTD (dark goldenrod). The missed structures of Apo-spike were shown in purple. S416 is shown as pink surface. (**E**) Top and side view of Apo-NTD (dark cyan), Apo-RBD3 (dark grey), S416-RBD3 (thistle) and S416-NTD (dark goldenrod). Arrows indicated remarkable conformational changes. (**F-H**) S416 (pink) and key residues are shown as sticks. S416-RBD3 (thistle), S416-NTD (dark goldenrod), Apo-RBD3 (dark grey) and Apo-NTD (dark cyan) are shown as cartoon. Arrows indicated remarkable conformational changes. The hydrogen bonds were shown as red dashed lines.


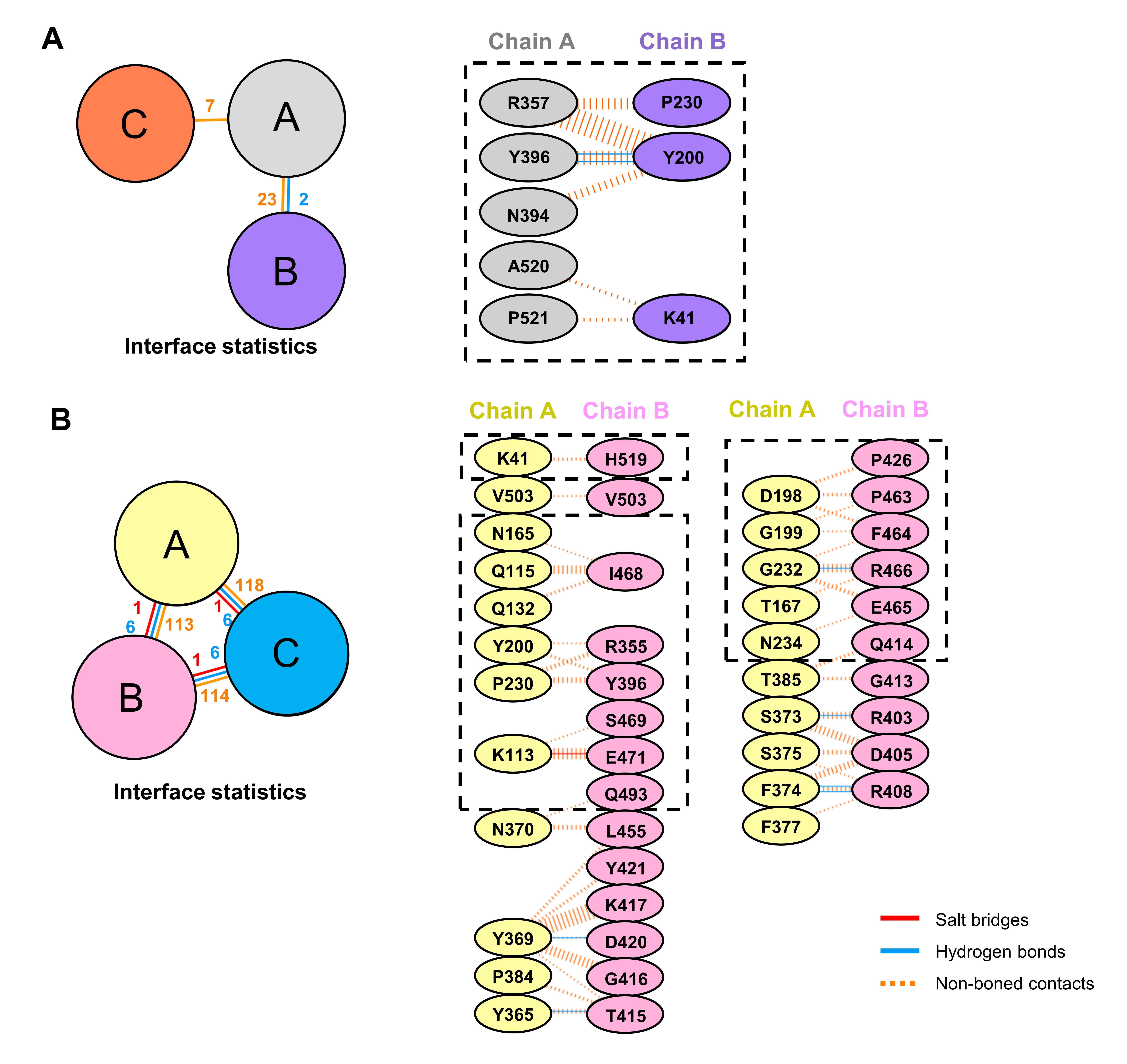


**Figure S7. Interface statistics of interactions for Apo and S416-bound spike trimer**. Interface statistics of interactions between different monomers and demonstration of specific interactions between RBD and NTD residues of chain A and chain B in apo **(A)** and S416-bound spike trimer (**B**). Interface interaction details were calculated through PDBsum ^39^. The RBD-NTD interdomain interactions are emphasized in black dashed boxes, while the remaining residues pairs display the RBD-RBD interdomain interactions.


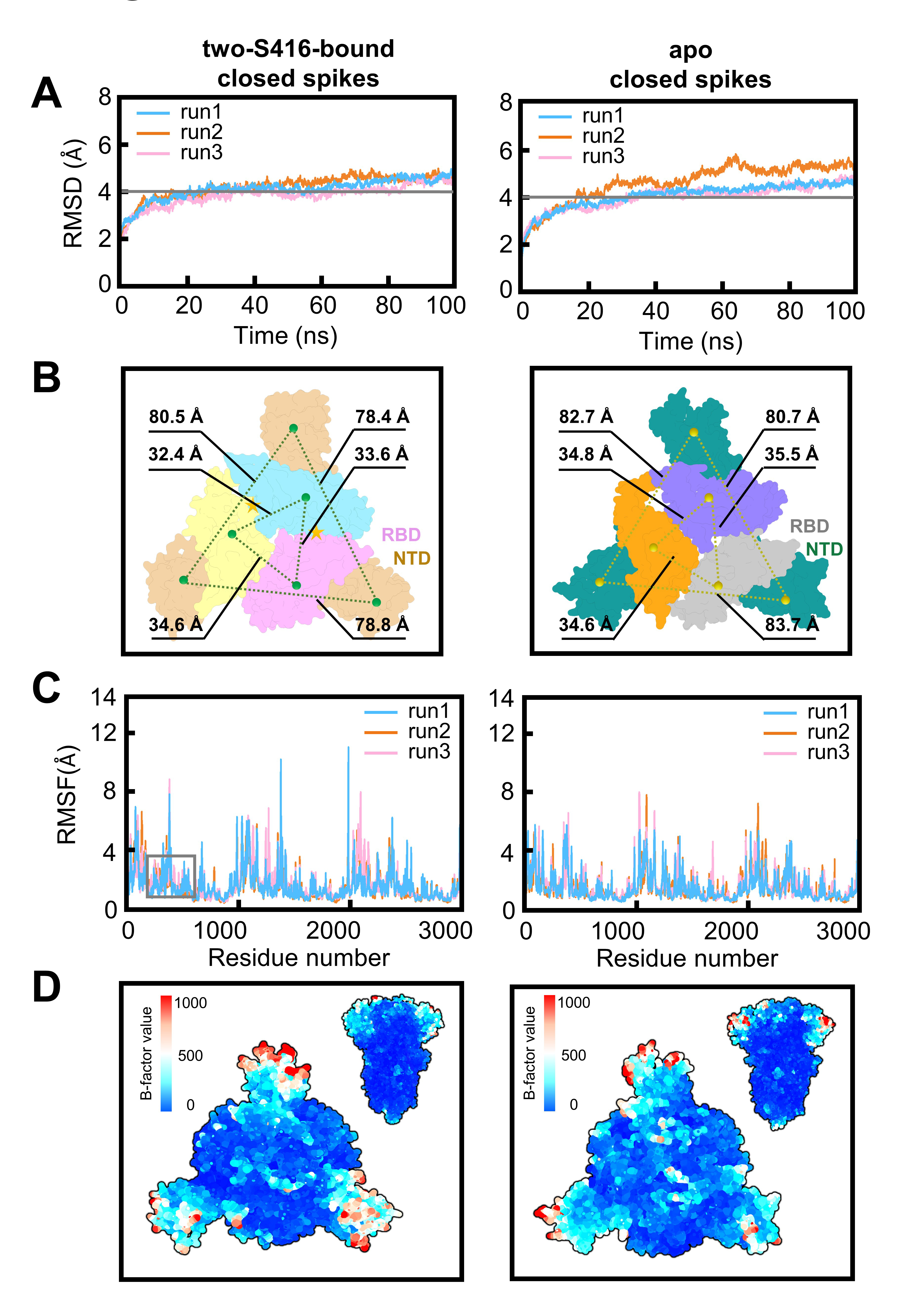


**Figure S8.** **Structural changes of S416-bound spike trimer studied by molecular dynamics simulation.** (**A**) The RMSD of apo closed, and two-S416-bound closed spike trimer during the 100 ns for three trajectories. (**B**) Analysis of the adjacent RBDs and NTDs core distance of the average conformation in apo closed and two-S416-bound closed spike trimer. Beads indicate the centra of mass of RBDs and NTDs, yellow pentagram indicates S416 binding site. (**C**) The RMSF of apo closed and two-S416-bound closed spike trimer during the 100 ns for three trajectories. (**D**) Visualization of the B-factor value-based heatmap of RBDs and NTDs of the representative conformation in apo closed and two-S416-bound closed spike trimer. Blue and red indicated smaller and bigger atomic fluctuations, respectively.


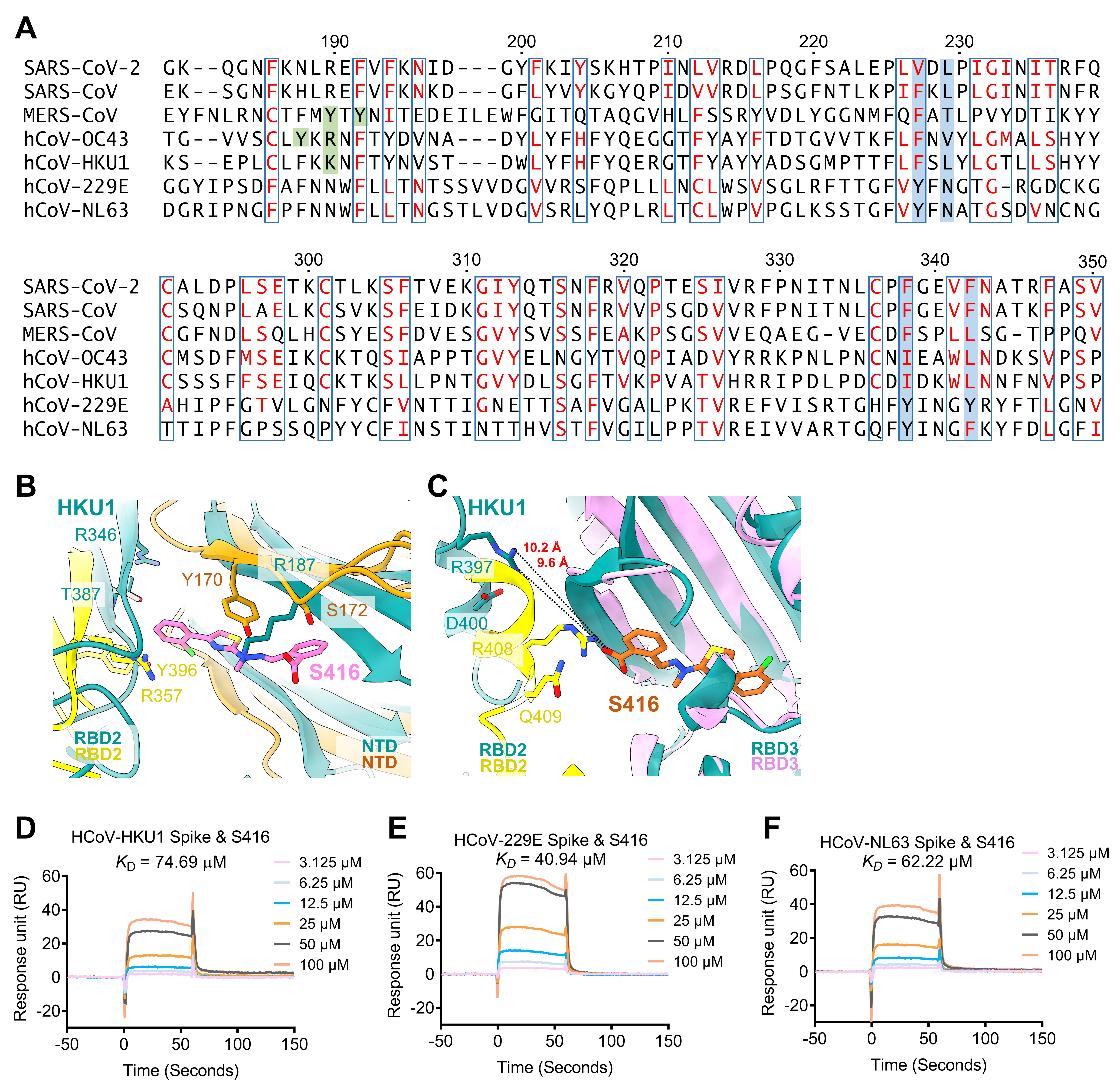


**Figure S9. Sequence and structural analysis of the human-infecting coronavirus.** (**A**) Alignments of the seven coronaviruses that can infect humans in the NTD-pocket and RBD-pocket. The conserved residues are highlighted in the composite binding pocket. Residues lining the hydrophobic pocket are underlaid (light blue). Residues positioned to interact with the S416 polar carboxyl group in the NTD-pocket are underlaid in green. Superimposition of the NTD (**B**) and RBD (**C**) of S416-bound SARS-CoV-2 (NTD, dark goldenrod; RBD2, yellow; RBD3, thistle) with those of the ligand free “apo” HKU1 (NTD, RBD2 and RBD3 in teal, PDBID: 5I08), respectively. (**D-F**) SPR analysis of the binding affinities for S416 with the spike protein from hCoV-HKU1, hCoV-229E and hCoV-NL63.

**Table S1. Cryo-EM data collection, refinement and validation statistics.**

| **Data collection and processing** | **Apo-spike protein** | **S416-spike protein** |
| --- | --- | --- |
| Magnification | 105,000 | 105,000 |
| Voltage (kV) | 300 | 300 |
| Electron exposure (e /Å) | 49.13 | 54.19 |
| Defocus range (pm) | -1.0 to -2.0 | -1.0 to -2.0 |
| Pixel size (Å) | 0.83 | 0.83 |
| Symmetry imposed | C1 | C1 |
| Initial particle images (no.) | 2,858,021 | 5,921,528 |
| Final particle images (no.) | 177,481 | 174,780 |
| Map resolution (Å) | 2.86 | 2.95 |
| FSC threshold | 0.143 | 0.143 |
| **Refinement** |  |  |
| Initial model used (PDB) | 6VYB | 7JJI |
| Map sharpening B-factor (Å^2^) | -50 | -50 |
| **Model composition** |  |  |
| Non-hydrogen atoms | 21479 | 25963 |
| Protein residues | 2867 | 3326 |
| **Average B factor (Å^2^)** |  |  |
| Protein | 57.52/287.46/125.32 | 47.14/147.84/73.02 |
| Ligand |  | 178.19 |
| **R.m.s. deviations** |  |  |
| Bond lengths (Å) | 0.003 | 0.004 |
| Bond angles (°) | 0.575 | 0.573 |
| **Validation** |  |  |
| MolProbity score | 1.87 | 1.66 |
| Clash score | 8.36 | 6.63 |
| **Ramachandran plot** |  |  |
| Favored (%) | 93.64 | 95.74 |
| Allowed (%) | 5.36 | 4.26 |

**Table S2. Primers and TaqMan probes used.**

| **Primer** | **Sequence (5’→3’)** |
| --- | --- |
| HKU-N-F | TAATCAGACAAGGAACTGATTA |
| HKU-N-R | CGAAGGTGTGACTTCCATG |
| HKU-N-P | GCAAATTGTGCAATTTGCGG |
| Berlin_E_F | ACAGGTACGTTAATAGTTAATAGCGT |
| Berlin_E_R | ATATTGCAGCAGTACGCACACA |
| Berlin_E_P1 | ACACTAGCCATCCTTACTGCGCTTCG |
